# Sexual reproduction of the placental brooder *Celleporella hyalina* (Bryozoa, Cheilostomata) in the White Sea

**DOI:** 10.1002/jmor.20943

**Published:** 2019-01-17

**Authors:** Uliana A. Nekliudova, Thomas F. Schwaha, Olga N. Kotenko, Daniela Gruber, Norbert Cyran, Andrew N. Ostrovsky

**Affiliations:** ^1^ Department of Integrative Zoology, Faculty of Life Sciences University of Vienna Vienna Austria; ^2^ Department of Invertebrate Zoology, Faculty of Biology Saint Petersburg State University Saint Petersburg Russia; ^3^ Core Facility Cell Imaging and Ultrastructure Research Faculty of Life Sciences, University of Vienna Vienna Austria; ^4^ Department of Palaeontology, Faculty of Earth Sciences Geography and Astronomy, University of Vienna Vienna Austria

**Keywords:** colonial aquatic invertebrates, life‐history, matrotrophy, oogenesis, placental analogue

## Abstract

The evolution of parental care is a central field in many ecological and evolutionary studies, but integral approaches encompassing various life‐history traits are not common. Else, the structure, development and functioning of the placental analogues in invertebrates are poorly understood. Here, we describe the life‐history, sexual colony dynamics, oogenesis, fertilization and brooding in the boreal‐Arctic cheilostome bryozoan *Celleporella hyalina*. This placental brooder incubates its progeny in calcified protective chambers (ovicells) formed by polymorphic sexual zooids. We conducted a detailed ultrastructural study of the ovary and oogenesis, and provide evidence of both auto‐ and heterosynthetic mechanisms of vitellogenesis. We detected sperm inside the early oocyte and within funicular strands, and discuss possible variants of fertilization. We also detail the development and functioning of the placental analogue (embryophore) in the various stages of embryonic incubation as well as embryonic histotrophic nourishment. In contrast to all known cheilostome placentas, the main part of embryophore of *C. hyalina* is not a single cell layer. Rather, it is a massive “nutritive tissue” whose basal part is associated with funicular strands presumably providing transport function. *C. hyalina* shows a mixture of reproductive traits with macrolecithal oogenesis and well‐developed placenta. These features give it an intermediate position in the continuum of variation of matrotrophic provisioning between lecithotrophic and placentotrophic cheilostome brooders. The structural and developmental differences revealed in the placental analogue of *C. hyalina*, together with its position on the bryozoan molecular tree, point to the independent origin of placentation in the family Hippothoidae.

## Graphical abstract

Histological sections of the female polymorphic zooids of *Celleporella hyalina* showing consecutive stages of embryonic growth and embryophore (placental analogue) development in the brood chamber (ovicell). Early embryo and placental analogue in the early stage are seen in the upper image. The bottom image shows advanced embryo that occupies most of the brood cavity. Well‐developed placental analogue is in the mid‐stage. Later on it can occupy almost half of the female zooid.

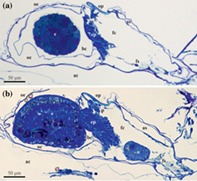

## 1 INTRODUCTION

The mode and timing of parental investment in developing progeny are among the most important aspects of sexual reproduction (Lodé, 2012; Pollux, Pires, Banet, & Reznick, 2009). In particular, parental care is a critical life‐history trait directly affecting offspring survival and, often, fitness (Avise, 2013; Clutton‐Brock, 1991; Royle, Smiseth, & Kölliker, 2012). Matrotrophy or extraembryonic nutrition (EEN), that is, the direct provisioning of nutrients from the parent to incubated youth, is one of the most effective modes of parental care, combining offspring protection and nourishment. Most studies on matrotrophy (and its most elaborate form, placentotrophy) have been undertaken on vertebrates (reviewed in Amoroso, 1968; Blackburn, 2005, 2015; Lombardi, 1998; Wooding & Burton, 2008; Wourms, 1981; Wourms, Grove, & Lombardi, 1988); its expressions among invertebrates remain largely unexplored.

The first comprehensive analysis of EEN across the animal kingdom revealed that this phenomenon is established or inferred in at least 21 of 33 animal phyla (Ostrovsky et al., 2016). This number significantly exceeds previous accounts and contradicts the traditional view that matrotrophy is infrequent among invertebrates (see Avise, 2013; Clutton‐Brock, 1991; Hogarth, 1976; Trumbo, 2012). Else, the analysis of the distribution and diversity of matrotrophic adaptations (both structural and physiological) in Animalia estimated 140–145 independent origins of this phenomenon (Ostrovsky et al., 2016).

Matrotrophy is associated with all known types of incubation chambers, or performed without them and using five nutritive modes: histotrophy, placentotrophy, oophagy, embryophagy and histophagy, of which the first and the second are the most widespread (Ostrovsky et al., 2016). Nutrient delivery and uptake are performed using secretion, active transport across membranes, facilitated diffusion, endocytosis (pino‐ and phagocytosis) as well as ingestion of parentally derived nutritive material and sometimes of germ and parental somatic cells. Overall, invertebrate matrotrophic adaptations are less complex structurally than in vertebrates (and chordates, in general), but they are extraordinarily diverse in respect to the sites, modes, mechanisms and structures involved. Despite the current progress in our understanding of this diversity, only few matrotrophic invertebrates have been studied ultrastructurally. This impairs comparative and evolutionary analyses.

The entirely colonial, lophotrochozoan phylum Bryozoa has the widest taxonomic distribution of placental analogues among aquatic invertebrates (Ostrovsky et al., 2016). Among three bryozoan classes, placentation is presumably characteristic to all representatives of Stenolaemata and Phylactolaemata, and is common in the class Gymnolaemata. The distribution patterns as well as the differences in the structure of incubation chambers, in the cell source, position and anatomy of the placental analogues in different clades indicate at least 23 independent origins of matrotrophy within Bryozoa. This makes this phylum an exceptional model to study trends in the evolution of matrotrophy in animals (Ostrovsky, 2013a, 2013b; Ostrovsky, Gordon, & Lidgard, 2009; Reed, 1991; Ryland, 1976).

The overwhelming majority of independent transitions to EEN occurred within the gymnolaemate order Cheilostomata. This type of nutrition occurs either in internal brood sacs or inside external calcified brood chambers—ovicells (Ostrovsky, 2013a). The opening of the ovicell is normally plugged by the specialized outgrowth of the membraneous wall of the fertile zooid (termed an ooecial vesicle) that in matrotrophic species bears an embryophore, that is, a placental analogue providing nourishment for the embryo. An active embryophore consists of hypertrophied epithelial lining and associated funicular tissue (Hughes, 1987; Moosbrugger, Schwaha, Walzl, Obst, & Ostrovsky, 2012; Woollacott & Zimmer, 1972, 1975). In the internal brooders, the entire wall of the brood sac becomes an embryophore. At present, placental analogues have been recorded in 21 cheilostome species belonging to 10 families (Ostrovsky, 2013a, 2013b; Ostrovsky et al., 2009), but only three species of two families were studied ultrastructurally (Hughes, 1987; Moosbrugger et al., 2012; Woollacott & Zimmer, 1975). Moreover, sexual reproduction in most placental bryozoans has been studied only fragmentarily (reviewed in Ostrovsky, 2013a).

This study focuses on the reproductive biology of the common boreal‐Arctic cheilostome *Celleporella hyalina* (Linnaeus, 1767). It demonstrates a prominent example of placentation due to its specialized sexually polymorphic zooids. Colonies of this species are simultaneous hermaphrodites comprising feeding autozooids and sexual male and female autozooidal polymorphs that are unable to feed. Embryos are brooded in the ovicells of the female zooids and are supplied by a well‐developed placental analogue. As females do not feed, the EEN is provided by the neigbouring autozooids via a transport system of funicular strands/cords connected via interzooidal communication pores (Hughes, 1987; Ostrovsky, 1998).

*C. hyalina* has been an object of extensive field and experimental studies (predominantly by Hughes with co‐authors) focusing on various aspects of fertilization and sex allocation (Bishop, Manríquez, & Hughes, 2000; Hoare & Hughes, 2001; Hoare, Hughes, & Goldson, 1999; Hughes, Manríquez, & Bishop, 2002; Hughes & Wright, 2014; Hughes, Wright, Carvalho, & Hutchinson, 2009; Hughes, Wright, & Manríquez, 2002; Hunter & Hughes, 1993, 1995; Hunter, Hughes, & Goldson, 1996; Manríquez, Hughes, & Bishop, 2001, 2002; Pemberton, Hughes, Manríquez, & Bishop, 2003). Another focus has been on life‐history traits, including growth and fitness, and their plasticity (Atkinson, Morley, & Hughes, 2006; Cancino, 1986; Cancino & Hughes, 1987, 1988; Eggleston, 1972; Hughes, 1989, 1992; Hughes & Hughes, 1986; Hughes, Manríquez, Bishop, & Burrows, 2003; Hughes, Manríquez, Morley, Craig, & Bishop, 2004). In contrast, only four morphological studies on the sexual reproduction of this species have been published. Hughes (1987) investigated the formation of the sexual zooids and ovicells, as well as fecundity, gametogenesis and brooding of *C. hyalina* from the Irish Sea using histological sections and scanning and transmission electron microscopy (SEM and TEM). The development and structure of the ovicells, along with certain aspects of oogenesis and embryonic incubation, were studied on the specimens from the White Sea by Ostrovsky (1998, 2013a, 2013b) using SEM and histological techniques. However, both oogenesis and placental nourishment, while providing a comparative basis for our study, were described rather superficially. Certain conclusions were only partially supported, calling for more detailed and broader research. The main focus of this study was on the ultrastructure of oogenesis and the development of the placental analogue along with its functioning on various stages of embryonic/larval growth. We also for the first time report the main life‐history traits of this bryozoan species in the White Sea, yielding an integral picture of its sexual reproduction.

## 2 MATERIALS AND METHODS

In the White Sea, colonies of *C. hyalina* (Linnaeus, 1767) range from the intertidal down to 137 m depth, encrusting various substrates, typically, algae (Gostilovskaya, 1978). We collected bryozoans on kelps (*Saccharina latissima* species‐complex) and red algae (*Odonthalia dentata, Phycodrys rubens, Coccotylus truncatus*) during the ice‐free period from 5–10 m depth by boat dredging and SCUBA‐diving near the Educational and Research Station ‘Belomorskaia’, Saint Petersburg State University (Chupa Inlet, Kandalaksha Bay, White Sea).

To study the life‐history, the random sampling was performed in 2012 and 2014 (Supporting Information Tables 1 and 2). Vast majority of the colonies were collected between May and September, 2014. Altogether the state of 1,003 colonies was examined using qualitative parameters, that is, colony shape and relative size, zooidal performance (feeding, budding and polypide degeneration), and presence of sexual polymorphs and embryos in them. Recording of these parameters in different months allowed recognition of main life‐history traits, number of generations, colony sexual dynamics and life‐span and timing of reproduction.

Alive colonies were photographed with a digital camera Leica DFC295 attached to a Leica M205C stereomicroscope.

For anatomical studies, colonies were collected in 2013 and 2015 (Supporting Information Table 3). They were fixed and decalcified in Bouin's fluid. After dehydration in ethanol series (30‐50‐70‐80‐90‐96%) they were embedded in resin (Epon 812), sectioned (2.0 μm thick) and stained by Richardson's stain by standard methods (Richardson, Jarrett, & Finke, 1960). Images were made with a Nikon DS‐Fi1 photocamera attached to a Leica DM2500 stereomicroscope. Altogether, ovaries from 78 zooids from five *C. hyalina* colonies were studied. Total preparations of some colonies fixed either in the Bouin's fluid or in 70% ethanol were made after dehydratation and embedding them in epon. They were photographed with a Leica DFC420 photocamera (Leica Microsystems, Wetzlar, Germany) attached to a Leica M205C stereomicroscope to estimate the colony size and the number of female polymorphs.

For ultrastructural studies of oogenesis and placentation 20 colonies were collected in 2013, 2016 and 2017. They were fixed in 2.5% glutaraldehyde (in 0.1 mol L^−1^ cacodylate buffer with 10% sucrose, pH 7.4) for 3 hr and subsequently rinsed three times in the buffer. Postfixation was done in a 1% solution of osmium tetroxide (OsO4) in the buffer solution for 1 hr followed by three rinses in the buffer. Decalcification involved several hours in 5% solution of EGTA in the buffer. After rinsing in the buffer, all colonies were dehydrated in a graded ethanol series (30‐50‐70‐80‐90‐96%) and in ethanol‐acetone mixtures with acetone (3:1–1:1–1:3) and subsequently embedded in epoxy resin (Agar LVR—Low Viscosity Resin). Resin blocks were sectioned using a Reichert UltraCut S microtome with Diatome Histo‐Jumbo and Diatome 35° Ultra diamond knives (Diatome, Bern, Switzerland). Altogether 36 female zooids from 20 colonies were sectioned. Ultrathin sections of 60 nm thickness were placed on the copper grids and contrasted with 2.5% gadolinium triacetate and 3% lead citrate. Sections were examined with a Zeiss Libra 120 transmission electron microscope (Zeiss, Jena, Germany) and photographed with a digital CCD Olympus Morada G2 (11 MP, in column) camera.

Characteristics of the sexual reproduction in the colonies collected in different years did not differ.

## 3 RESULTS

### 3.1 Life‐history and colony sexual dynamics

**Figure 1:**
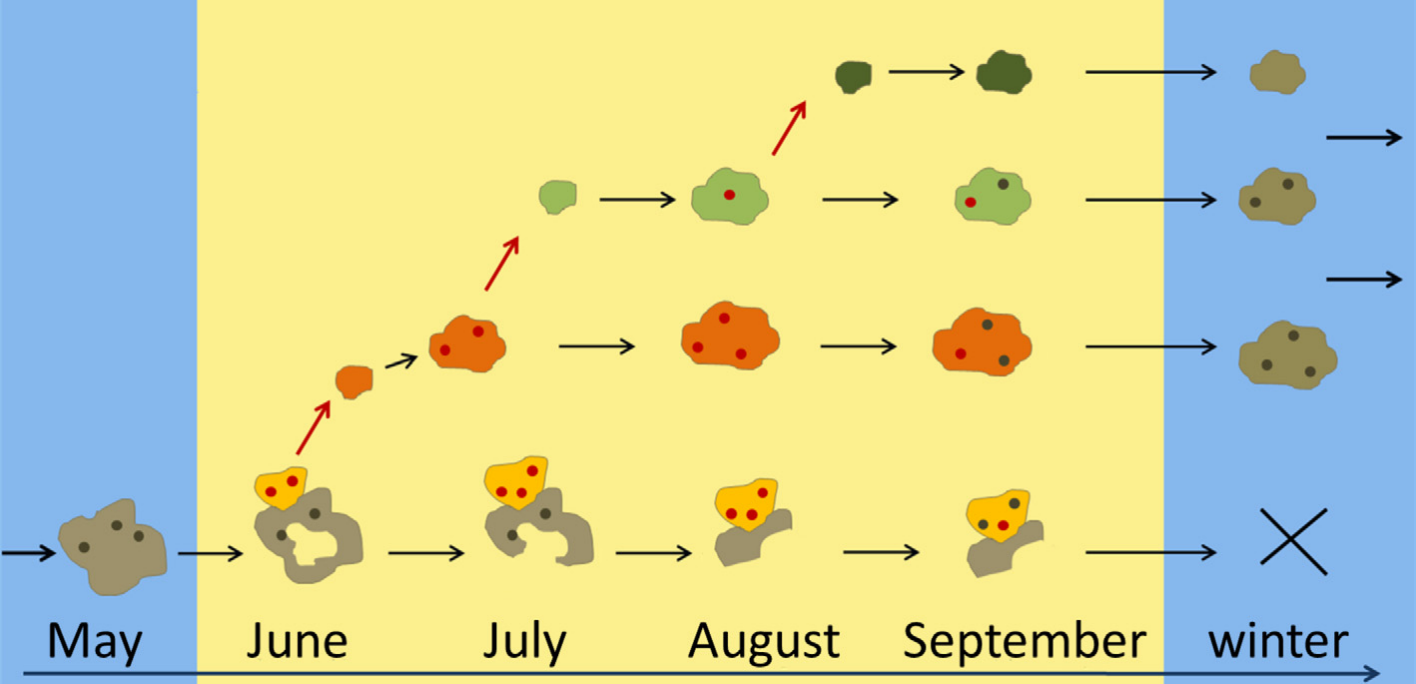
*C. hyalina,* scheme of the life‐cycle in the White Sea. Colonies of the overwintered (parental) generation shown in the lower line, yellow parts represent newly grown areas. Summer generations are above the parental one. Ovicells with embryos shown as red circles, empty as black ones

**Figure 2:**
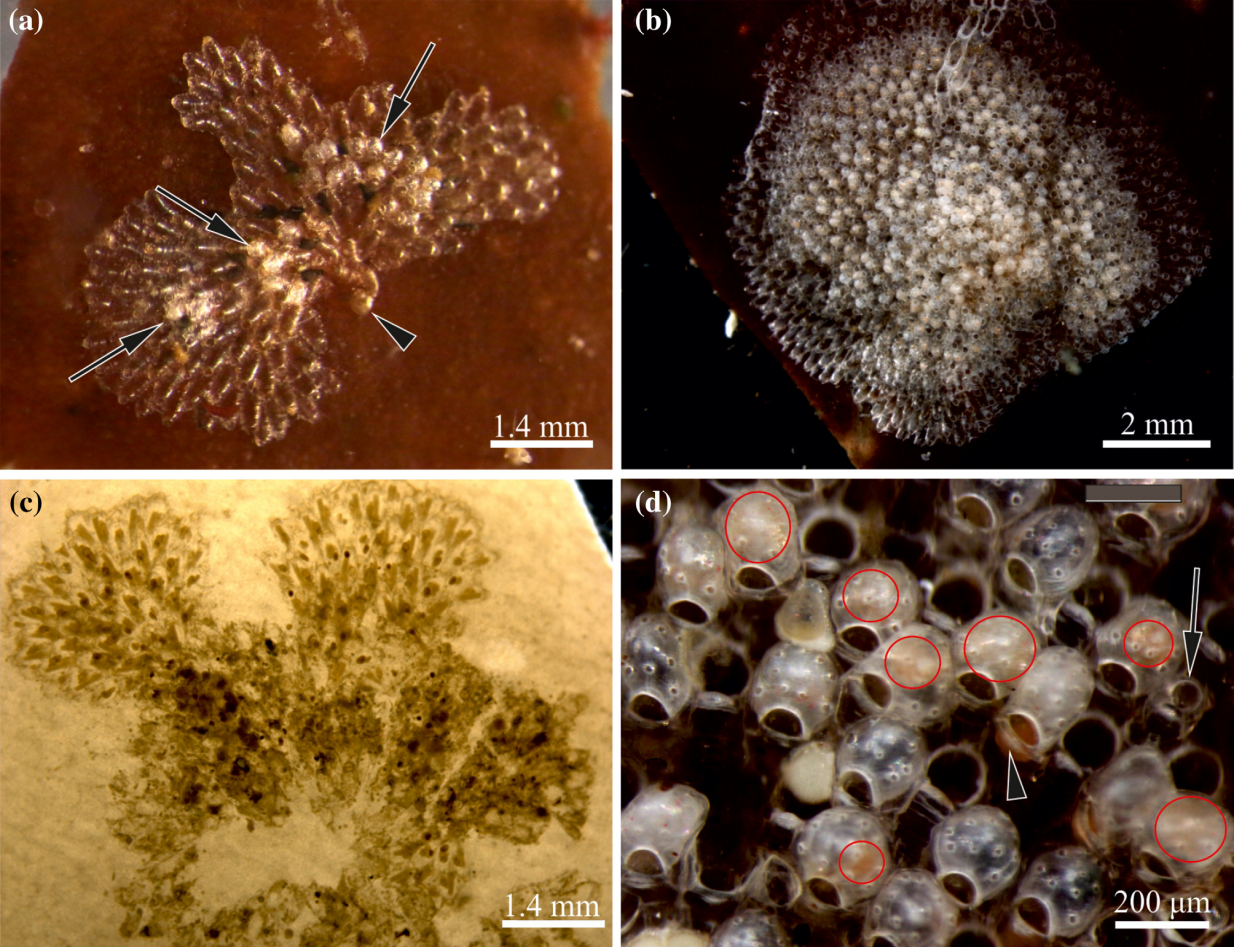
*C. hyalina,* general view of the colonies. (a) Young colony of the second generation with three groups of the frontal female polymorphs (arrows) and ancestrula (arrowhead) (living specimen collected in June 2014). (b) Full‐grown mature colony of the second generation consisting of the basal autozooidal layer (visible on the colony periphery) and frontal layer of mostly female polymorphs (living specimen collected in the end of July 2017). (c) Overwintered colony of the parental (first) generation with lobate outline and destroyed central part (colony decalcified) (collected in June 2014). (d) Close‐up of the frontal colony surface showing male dwarf polymorph (arrow) and dwarf females with ovicells—empty and containing growing embryos of various sizes (outlined by red lines). Ripe oocyte ready for oviposition is shown by arrowhead. Larger autozooidal apertures are interspersed between female polymorphs

In the studied population, two age groups were easily distinguished by their appearance: old overwintered colonies formed in the previous year/ice‐free period and young colonies formed during the current year (Figure 1, Supporting Information Tables 1 and 2). The former were characterized by the low transparency of their skeleton, the frequent presence of epibiotic microalgae, infusorians and hydrozoans, and the irregular shape of the colony consisting of the old deteriorating and new budding parts. They were recorded from May to September. Young colonies were patch‐like, with more transparent zooidal walls without microfoulers. They were encountered from June to September being represented by two or, highly likely, three generations. Larval production occurred from June to September and involved both, old and young colonies (Figures 1, 2).

Overwintered colonies, collected in May, were inactive without any sign of feeding, budding or reproduction. Only brown bodies (degenerated polypides) were visible through the zooidal walls in some zooids. These overwintered colonies resumed a peripheral growth and began or resumed reproduction in June, giving rise to the small colonies of the daughter (second) generation that appear on algae (Figures 1, 2, Supporting Information Table 2). During summer, their old (overwintered) areas were gradually destroyed (mainly in the colony center), but their newly formed parts (often resembling peripheral subcolonies; Figure 2c) continued growth and larval production until late August, and possibly, early September. We did not find evidence of their second hibernation and suppose them to die in winter.

Young colonies appeared in the studied population from June to late September (Figures 1, 2a, Supporting Information Tables 1 and 2). They were actively growing, representing the second and third (and, possibly fourth) colony generations. The earliest daughter colonies already started reproduction in mid‐June (sometimes consisting of only 10 autozooids and 1–4 female polymorphs). They provided a beginning of the third generation established in late June that, in turn, should start reproduction in July thus giving the fourth generation. The number of generations is inferred from the peaks of young colonies establishment (corresponding decrease of young colonies mean size and increase of percentage of non‐reproducing colonies among them) and the presence of small reproducing colonies from mid‐June till the end of September (Supporting Information Table 2). Throughout September, most colonies of the ‘summer (young) generations’ grew and reproduced, while others were apparently preparing for dormancy: a few colonies were found that did not grow or feed at the end of that month, and their autozooids possessed either brown bodies or degenerating polypides. Some of them might be dead.

On establishment, the young colonies of *C. hyalina* are sterile and consist of one layer of autozooids further added by a few additional basal male autozooidal polymorphs. Frontal budding of both, male and female sexual polymorphs, changes male colonies to hermaphrodites (Figure 2d). Sperm production ends earlier, making colonies female at the end of the reproductive period. In autumn, female gonads are also resorbed, and colonies become sterile again. Overwintered colonies first resume budding of basal autozooids, followed by frontal sexual zooids, thus repeating the same sequence as in young colonies. No repeated establishment of the ovaries in the overwintered female zooids was detected, and their ovicells did not contain embryos.

### 3.2 Ovary: Development and structure

**Figure 3:**
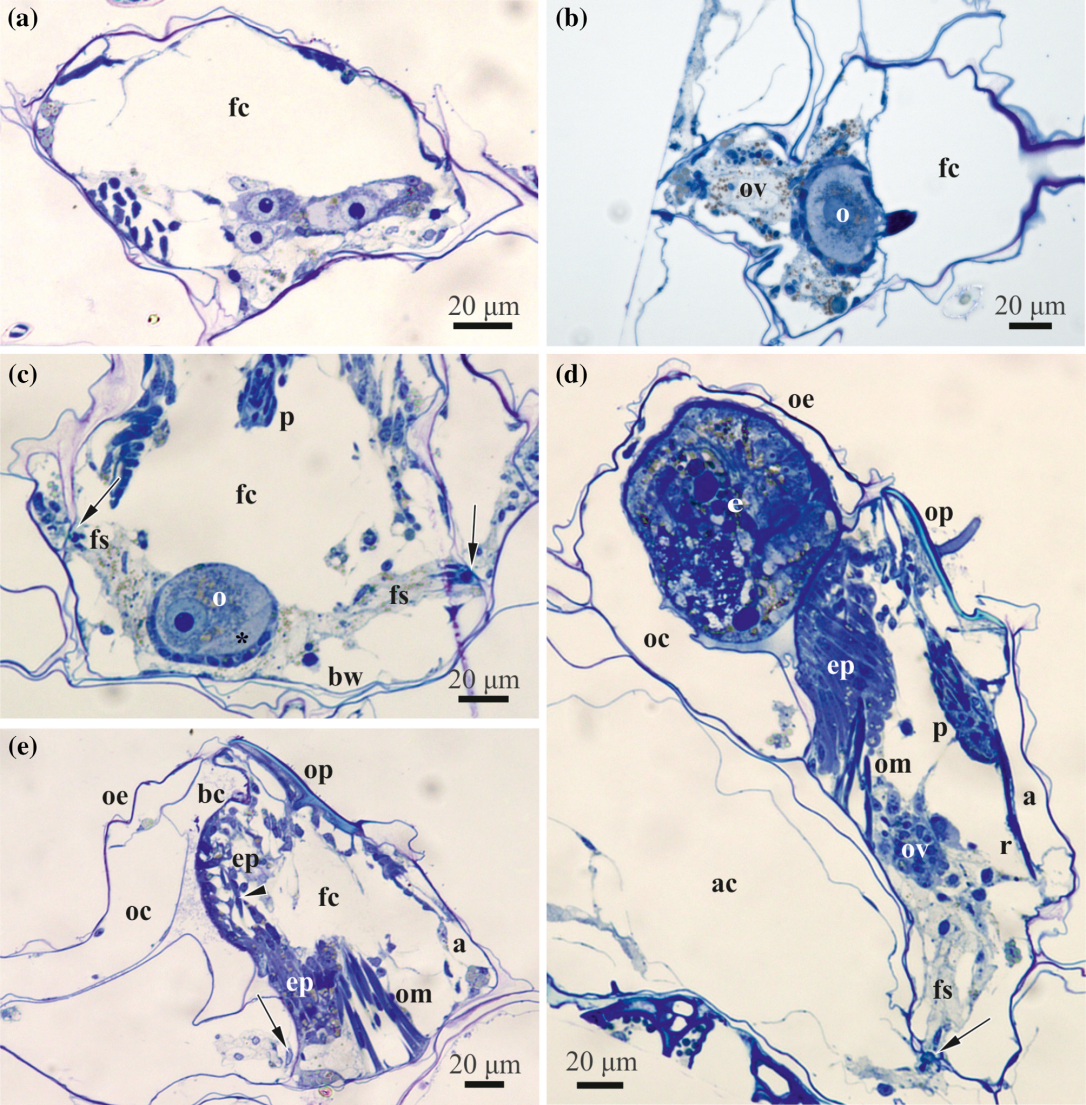
*C. hyalina,* histological sections of the female polymorphic zooids. (a) Cross‐section of the forming zooid with young ovary with previtellogenic oocyte doublet (two larger cells) and oogonium suspended on the funicular cords just above basal zooidal wall. (b) Oblique section of the female zooid with ovary of irregular shape consisting of three loose ‘lobes’ of mesothelial cells and follicle containing early vitellogenetic doublet (nurse cell is not seen). Yolk granules are visible in the oocyte as well as mesothelial cells of the ovary. (c) Cross‐section of the female zooid with early vitellogenic doublet (nurse cell marked by asterisk) in the ovary approached by two funicular strands that are connected to communication pores (arrows). Follicle consists of cuboidal and squamose cells. (d) Longitudinal section of the female zooid with ovary (germ cells are not seen), embryophore that is developed in association with the distal zooidal wall and embryo in the ovicell. Arrow shows communication pore connecting female polymorph with basal autozooid. Funicular strand approach the pore that is plugged by the pore‐cell complex. (e) Longitudinal section of the female zooid with empty ovicell (to the left, collapsed after decalcification) after larval release. Embryophore is in degenerating state. Arrowhead shows a muscle of the distal zooidal wall, arrows points to the communication pore connecting female polymorph with ooecium. Abbreviations: a = ascus; ac = coelom of basal autozooid; bc = brood cavity; bw = basal wall of female zooid; ep = embryophore; fc = coelom of female zooid; fs = funicular strand; o = oocyte; oc = coelom of ooecium; oe = ooecium (protective outfold of the ovicell); om = opercular muscles; op = operculum; ov = ovary; p = rudimentary polypide; r = retractor muscle of polypide

**Figure 4:**
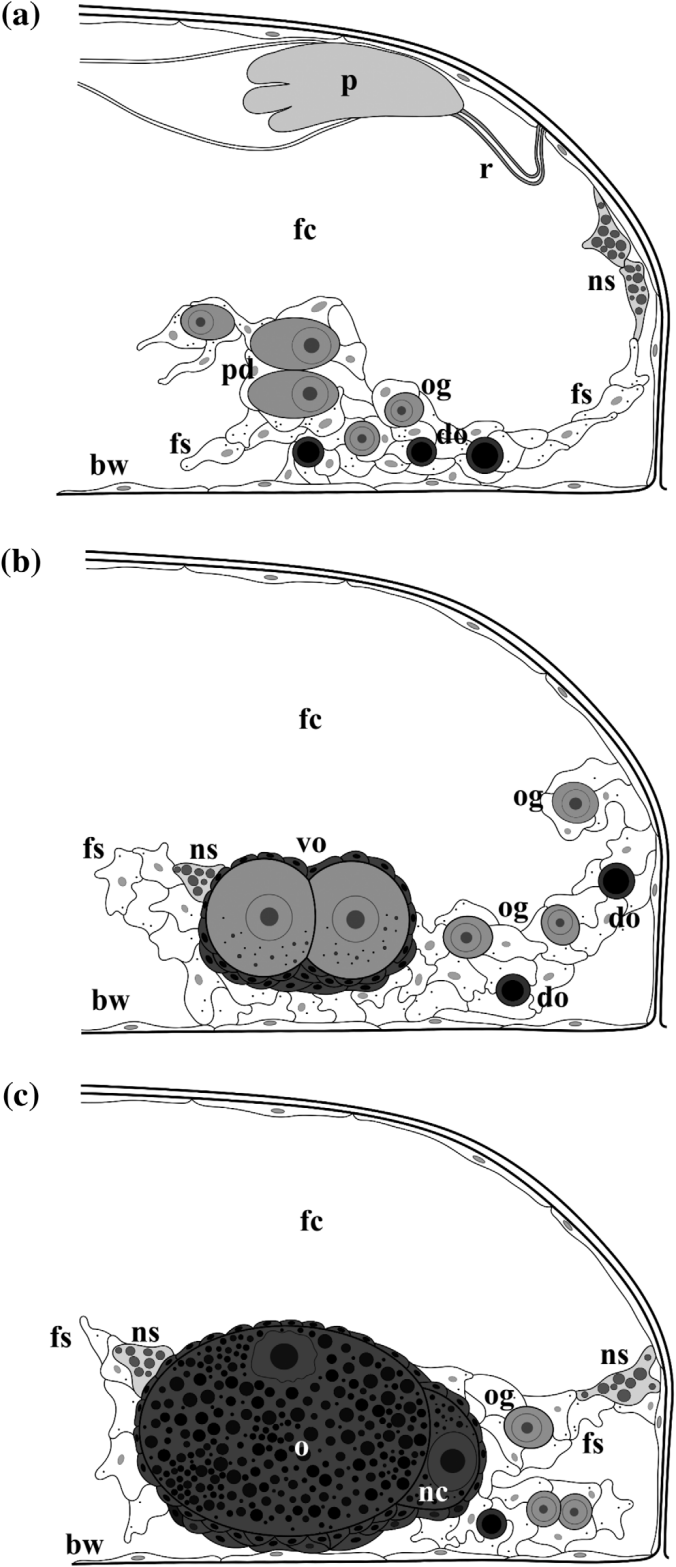
*C. hyalina,* schemes of the ovary development and major stages of oogenesis. (a) Young ovary with previtellogenic oocyte doublet and several round oogonia (some degenerating) suspended on the funicular cords just above basal zooidal wall; germ cells are surrounded by mesothelial cells. (b) Ovary with several oogonia surrounded by mesothelial cells and a follicle containing early vitellogenetic doublet. (c) Mature ovary with a follicle containing mature vitellogenetic doublet (yolk granules are visible in the oocyte as well as nurse cell), early doublet and oogonia (also degenerating). In (b) and (c) ovary is situated on the basal zooidal wall. Follicle cells are dark‐grey. Abbreviations: bw = basal wall of female zooid; do = degenerating oogonia; fc = coelom of female zooid; fs = funicular strand; nc = nurse cell; ns = nutrient‐storage cell; o = oocyte; og = oogony; ov = ovary; p = rudimentary polypide; pd = previtellogenic oocytic doublet; r = retractor muscle; vo = vitellogenic doublet

The earliest germ cells (oogonia) were detected in the young female polymorphic zooids with developing ovicells. They were round or oval, being distinguished from somatic cells due to their markedly larger size (10.0–23.3 × 13.3 μm). The division of the oogonium results in either a pair of oogonia (soon separated from each other) or an early oocyte doublet whose cells are interconnected via a cytoplasmic bridge (see below). Early female gonads contained 2–5 non‐paired oogonia and/or one (either oogonial or early oocyte) doublet in our material. Ovaries always had an irregular shape and were suspended in the zooidal coelomic cavity on funicular cords or, sometimes, positioned on the epithelial lining of the zooidal wall (Figures 3 and 4). Germ cells were surrounded by a thin layer of small flattened mesothelial cells (Figures 3a and 4a). One of the funicular cords is connected via a communication pore to the underlying autozooid (Figure 3d).

**Figure 5:**
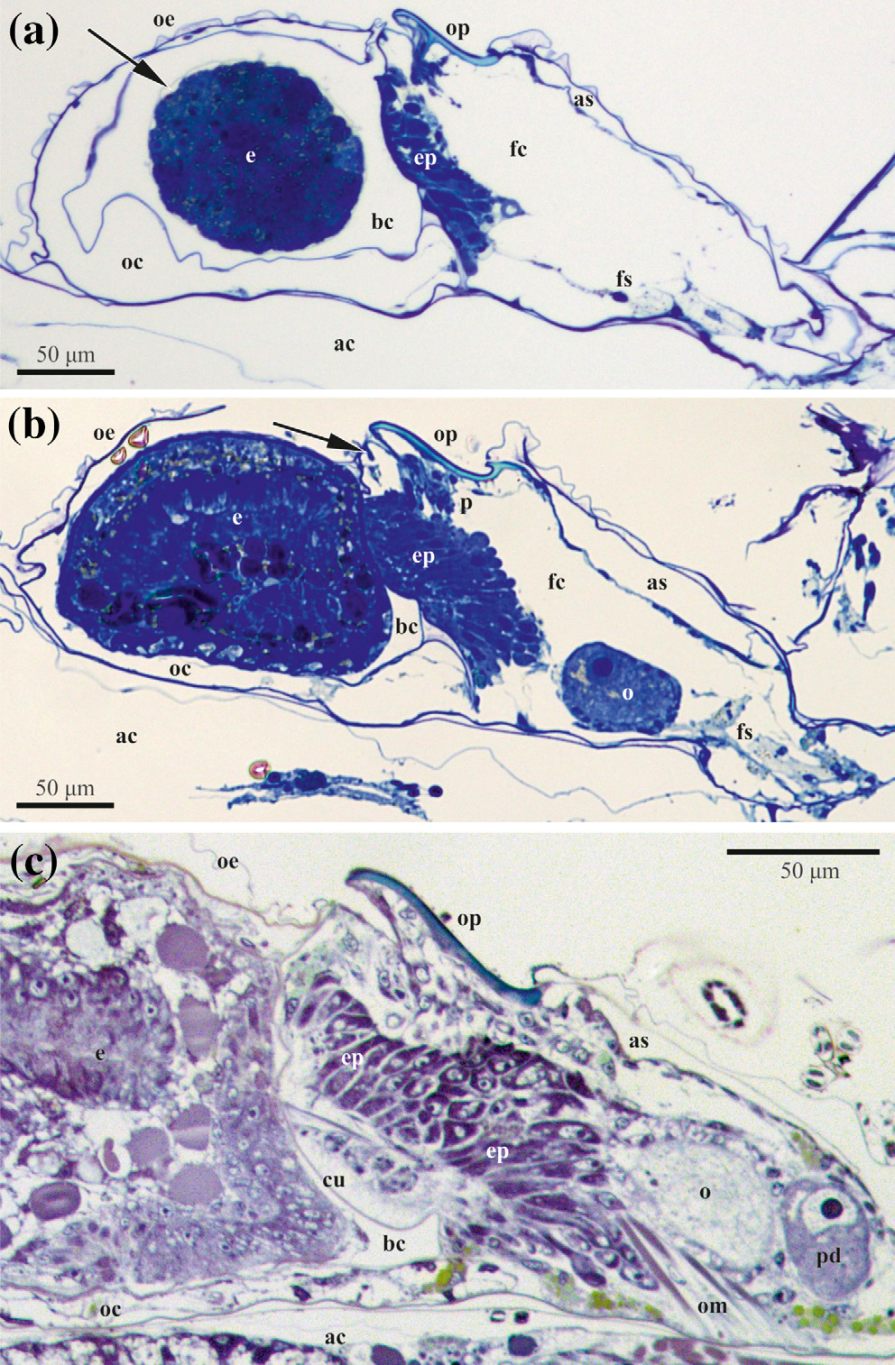
*C. hyalina,* longitudinal histological sections of the female polymorphic zooids showing stages of embryophore and embryo development. (a) Female polymorph with early embryo in the ovicell. Placental analogue (embryophore) is in the early stage of its development (arrow shows fertilization envelope surrounding embryo that partially occupies the brood cavity); ovary is not in the plane of sectioning. (b) Advanced embryo occupying most of the brood cavity. Embryophore is well‐developed. Ovary with the early vitelogenic oocyte is in the right part of the female zooid (its nurse cell is out of the section plane). Sclerite is clearly seen in the upper part of the distal zooidal wall in (a) and (b, shown by arrow). (c) Early larva in the ovicell. Embryophore occupies almost half of the female zooid; vitellogenic and previtellogenic oocyte doublets are seen in the ovary in right part of zooid. In all zooids embryophore is developed in association with the distal zooidal wall plugging the entrance to the brood chamber. Abbreviations: a = ascus; ac = coelom of basal autozooid; bc = brood cavity; cu = cuticle of distal zooidal wall; e = embryo; ep = embryophore; fc = coelom of female zooid; fs = funicular strand; o = oocyte in ovary; oc = coelom of ooecium; oe = ooecium (protective outfold of the ovicell); om = opercular muscles; op = operculum; p = rudimentary polypide; pd = previtellogenic doublet

Older developing ovaries contained up to 25 female cells, including solitary oogonia and 0–8 oogonial and/or early oocyte doublets (the two being indistinguishable at that time) (Figure 4b). Mature female gonad usually had one vitellogenic and one previtellogenic oocyte doublet (Figures 4c and 5c), as well as 5–6 oogonia (solitary or in doublets, although some could be the early oocyte doublets). Many sectioned female zooids, however, contained only one oocyte doublet in the mature ovary.

The fully formed female zooid contains a mature ovary that is suspended in the coelom on the funicular cords in the proximobasal part of the cystid. The ovary has an irregular shape and usually consists of a loose group of young female cells (oogonia and early oocytes) surrounded by mesothelial cells and 1–2 follicles with one oocyte doublet (vitellogenic and/or previtellogenic) each (Figures 3b,c and 5c). When containing a large mature oocyte, the follicle is often (but not always, see Figure 5c) positioned on the basal zooidal wall (Figure 4c) whereas the rest of the ovary is suspended in the zooidal cavity.

**Figure 6:**
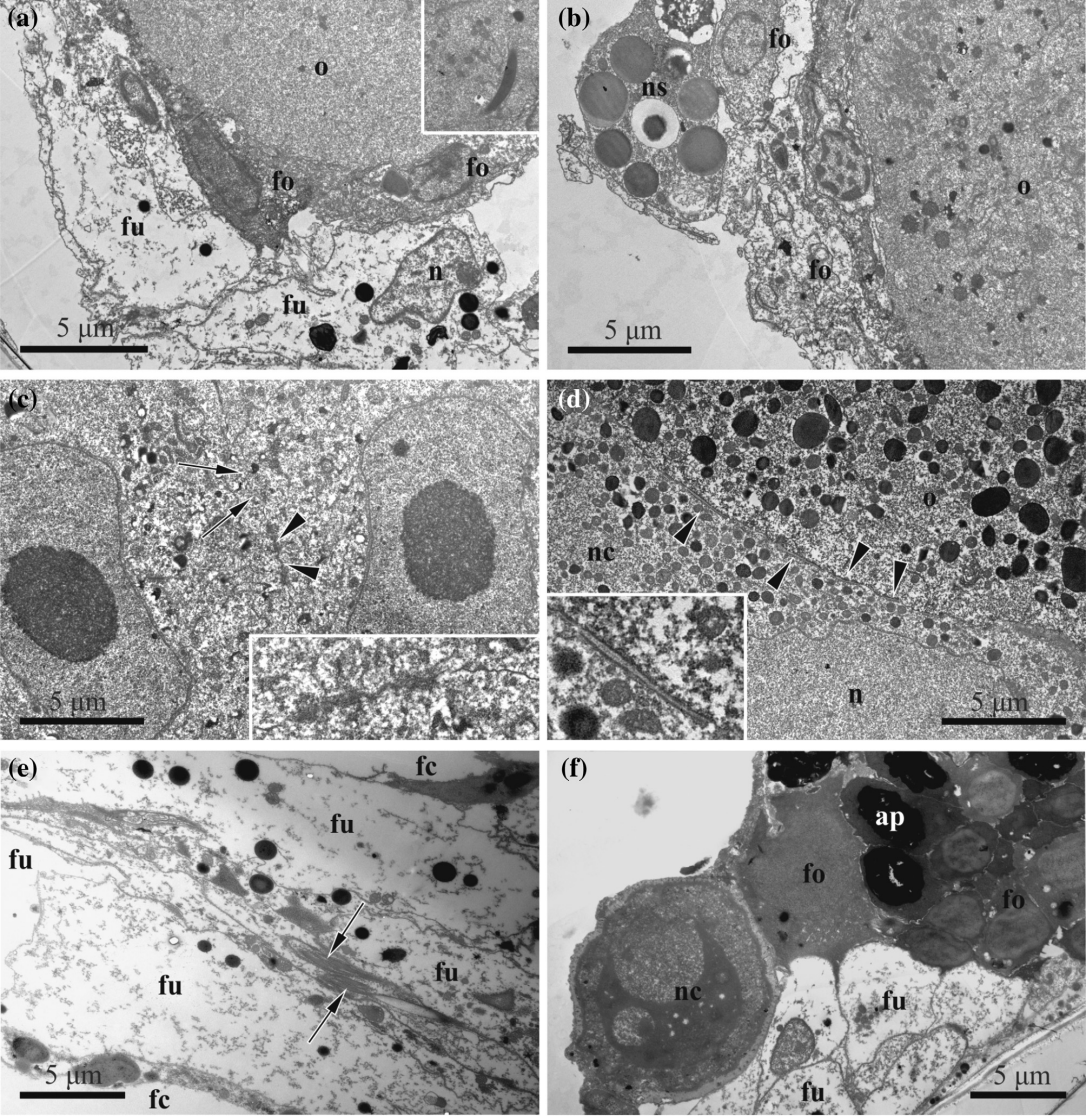
*C. hyalina,* early stages of oogenesis and fertilization (TEM). (a) Previtellogenic oocyte enveloped by follicular cells and funicular strand connected with the ovary; insert: Sperm nucleus in the cytoplasm of the same oocyte found in the other section plane. (b) Early vitellogenic oocyte enveloped by follicular cells; nutrient storage cell is seen on the ovarian wall. (c) Early oocytic doublet connected by complex cytoplasmic bridge: Membrane‐less areas enabling passage of the cytoplasm between the cells shown by arrows, and tight junction is indicated by arrowheads and shown in the insert; early small yolk granules are visible. (d) Early vitellogenic oocyte and its nurse cell connected by adherens junctions (shown by arrowheads and in the insert); nurse cell contains smaller and less yolk granules than the oocyte. (e) Two spermatozoa (arrows) between funicular cells. (f) Nurse cell degenerating outside the ovary (it is surrounded by the fertilization membrane with some microvilli embedded in it); degenerating follicle cells are visible too. Abbreviations: ap = autophagosome; fc = coelom of female zooid; fo = follicle cells; fu = funicular cells; n = nucleus; nc = nurse cell; ns = nutrient‐storage cell; o = oocyte

**Figure 7:**
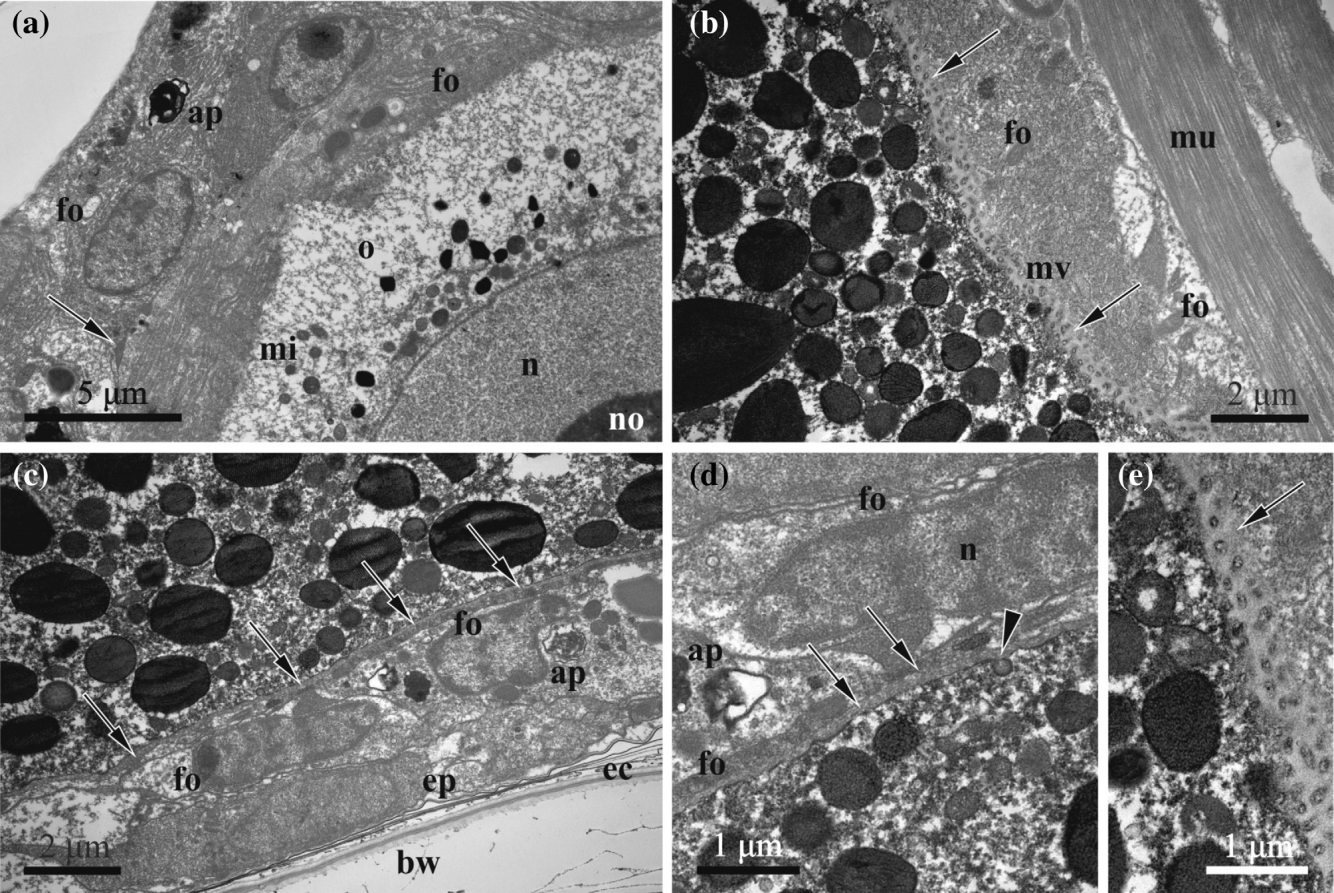
*C. hyalina,* vitellogenesis (TEM). (a) Part of the early vitellogenic oocyte enveloped by follicular cells with strongly developed RER; small yolk granules and mitochondria are visible close to the nuclear membrane (intercellular space filled with electron‐dense matrix shown by arrow). (b) Peripheral area of vitellogenic oocyte with microvilli embedded in the fertilization membrane (shown by arrows). (c) Partial view of the ovary situated on the basal zooidal wall; smooth plasmalemma of vitellogenic oocyte surrounded by fertilization membrane (shown by arrows). (d) Enlarged view of the same oocyte showing clathrin‐coated pit (arrowhead) and fertilization membrane (arrows). (e) Close‐up of the microvillous area of the oocyte (fertilization membrane shown by arrow). Abbreviations: ap = autophagosome; bw = basal zooidal wall; ec = ectocyst; ep = epithelial cells of the body wall; fo = follicle cells; mi = mitochondria; mu = muscle bundle; mv = microvilli; n = nucleus; no = nucleolus; o = oocyte

The follicle lining of the growing oocytic doublet is developed from the mesothelial cells surrounding oogonia and early oocytes (Figure 3a). In the early follicle with a previtellogenetic doublet, its cells are flattened, usually forming 1–2 layers, often with overlapping ends that sometimes form complex interdigitating areas (Figure 6a,b). Their either electron‐translucent or dense cytoplasm already contains an active nucleus, abundant free ribosomes and autophagosomes. At a more advanced stage, the follicle consists of cuboidal (in its basal part), flattened (forming lateral walls) and squamous cells (in the upper part; Figures 3c and 5b), forming 1–2 layers. Follicles with a large vitellogenetic oocytic doublet consist predominantly of flattened and squamous cells situated in 1–3 layers; thicker cells are present in the basal part. No subovarian zone is visible (Figures 4c and 5c). During vitellogenesis the cytoplasm of different follicular cells is of various electron density, and contains a large oval nucleus, numerous free ribosomes and abundant cisternae of rough endoplasmic reticulum (RER), single or stacked. Numerous mitochondria, Golgi complexes, protein platelets with homogeneous content and autophagosomes, often irregularly‐shaped, were also recorded (Figure 7). A few small intercellular spaces were visible between the follicle cells. These spaces were filled with an electron‐dark substance, possibly secreted material (Figure 7a).

The funicular strands approach the ovary from all sides (Figures 3a,c,d, 4, 5b, and 6a,f). Cells of the funicular strands have an irregular or elongated shape, sometimes with prominent processes, and a large lobate nucleus. The electron‐translucent cytoplasm is mainly empty, containing some free ribosomes, single RER cisternae, rare mitochondria, small lipid droplets and some other inclusions (Figure 6a,e,f). Sperm were recorded twice inside funicular cords (Figure 6e), close to the ovary. Resorbing oogonia and/or early oocytes—dark‐stained on histological sections and electron‐dense at fine sections—were often detected on the ovary periphery. Large cells with a cytoplasm filled with numerous protein platelets and various vacuoles (nutrient‐storage cells) were often detected in different parts of female zooids, including the follicle surface and epithelial lining of the zooidal wall (Figure 6b).

### 3.3 Oogenesis

Cells of the previtellogenic oocyte doublet are indistinguishable from each other (Figures 3a and 4a) unless a sperm nucleus is visible in one of the siblings (during our study such a nucleus was detected once in the cytoplasm of an early vitellogenic oocyte, Figure 6a, insert). Siblings are roundish (11.6–43.3 × 10.0–30.0 μm) and contain a large nucleus with slightly convoluted membrane (8.3–20.0 × 8.3–16.6 μm) and a large nucleolus (average diameter 5.3 μm). Their cytoplasm is electron‐dense, containing numerous free ribosomes, some mitochondria and single RER cisternae. Moreover, single cisternae of the smooth ER and Golgi apparatus were detected.

In early vitellogenesis, siblings are still of the same shape, size and have a similar ultrastructure. Small yolk granules (lipid droplets and protein platelets) begin to form in both cells (Figures 4b and 6c). The oolemma is smooth or slightly convoluted (Figures 6b and 7a). Short branchless microvilli appear at the apical hemisphere of the vitellogenic oocyte, further spreading to its vegetal pole and to the nurse cell. Mitochondria and single cisternae of RER in the oocyte and its sibling considerably increase in number at this stage. The cytoplasmic bridge, connecting cells of the doublet, is complex. It consists of several membrane‐less areas enabling passage of the cytoplasm between the cells, and of the oolemma with tight junctions (Figure 6c). The siblings are also connected by the adherens junctions (Figure 6d).

During vitellogenesis the oocyte enlarges 37.5‐fold (diameter range 26.6–110.0 × 16.6–50.0 μm), and its nucleus (13.3–26.6 × 11.6–20.0 μm) moves to its animal hemisphere (Figures 4b and 5b). The nucleus has a slightly convoluted membrane and a large spherical nucleolus (6.6–16.6 μm) sometimes containing electron‐translucent vacuoles. A few small additional nucleoli may be present. Numerous yolk granules fill the cytoplasm of the mature oocyte. Lipid droplets that are considerably less numerous and generally smaller than the protein platelets are brownish in the histological sections, whereas protein platelets are stained deep blue (Figure 3a,b,c). In the TEM images, the membrane‐bound protein platelets are grayish or dark‐gray, often showing paracrystalline structure, and the lipid droplets are black. Both, droplets and platelets, can be round or oval, and platelets sometimes angular (Figures 6d and 7). Beyond the yolk, mitochondria and free ribosomes are the most numerous organelles. Golgi complexes and RER cisternae become less prominent in the final stages of vitellogenesis.

The nurse cell grows less conspicuously (diameter range 20.0–46.6 × 16.6–33.3 μm), and its large nucleus (diameter range 13.3–26.6 × 10.0–23.3 μm) with non‐convoluted membrane occupies most of the cell volume (Figures 4c and 6f). Its cytoplasm has a lower electron density than in the oocyte and contains few small lipid droplets and protein platelets (Figure 6d). Both cells of the late vitellogenic doublet are covered by microvilli alternating with areas of smooth oolemma (Figure 7b–e). Microvilli are embedded in a thick fertilization membrane that is also visible in a narrow slit‐like space between the smooth areas of the oolemma and adjoining follicular cells. Clathrin‐coated pits were detected in the oolemma directly below this slit‐like space (Figure 7d).

During the reproductive season, each female polymorph of *C. hyalina* sequentially produces several macrolecithal isolecithal oocytes, but only one vitellogenetic doublet is visible in the ovary at the same timе. After ovulation, the mature oocyte is transferred into the ovicell where meiosis, karyogamy and embryogenesis occur, while the nurse cell and some follicle cells degenerate (Figure 6f). Growth of the next oocyte doublet follows the ovulation of the previous one, and the next vitellogenic oocyte develops in the ovary of the same female zooid simultaneously with embryogenesis (Figure 5b,c).

### 3.4 Development of the placental analogue and changes in the embryonic epithelium during incubation

**Figure 8:**
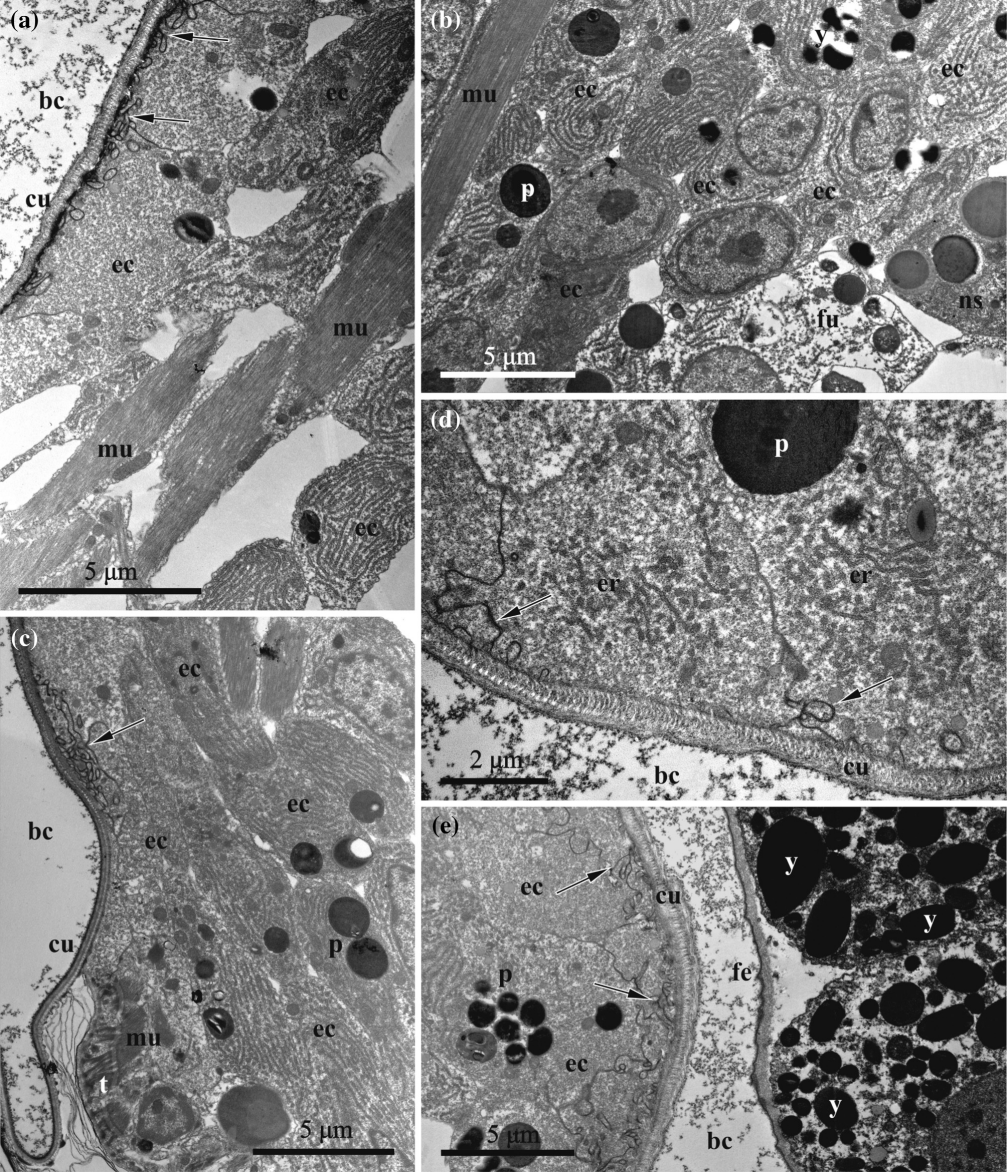
*C. hyalina,* embryophore at the early stage of embryonic incubation (TEM). (a) Part of the embryophore adjacent to the cuticle, showing loose pattern of the cell placement with large intercellular spaces; RER in the basally placed cells is more developed. (b) Basal part of the same embryophore with adjacent funicular cells and nutrient‐storage cell. (c) Compact group of nutritive cells in the lower part of the embryophore; attachment of the muscle bundles of the distal zooidal wall to the calcified skeleton (via tonofilaments) is visible in the left lower corner; (d) Apical parts of the embryophore (nutrient) cells adjacent to the cuticle; forming RER and large yolk granule are visible. (e) Early embryophore (to the left) close to the early embryo surrounded by fertilization envelope. Flocculent material in the brood cavity is visible in (a), (d) and (e), and protein(?) platelets in the nutritive cells in all images. Apical infoldings of the nutritive cells are indicated by arrows. Abbreviations: bc = brood cavity; cu = cuticle of the distal zooidal wall; ec = embryophore (nutritive) cells; er = endoplasmic reticulum; fe = fertilization envelope; fu = funicular cells; mu = muscle bundles of the distal zooidal wall; ns = nutrient‐storage cell; p = protein(?) platelet; t = tonofilaments; y = yolk granule

Each female polymorph containing an ovary is associated with the brood chamber (ovicell) consisting of the spherical protective capsule (ooecium) enveloping the brood cavity, and membranous distal wall of the female zooid plugging the entrance to this cavity. Female polymorphs had 2–4 tentacles and no digestive tract. Polypide retractor and occlusor muscles of the operculum as well as ascus with parietal (dilator) muscles and muscles of the distal wall are well‐developed (Figures 3, 5, and 8b,c).

**Figure 9:**
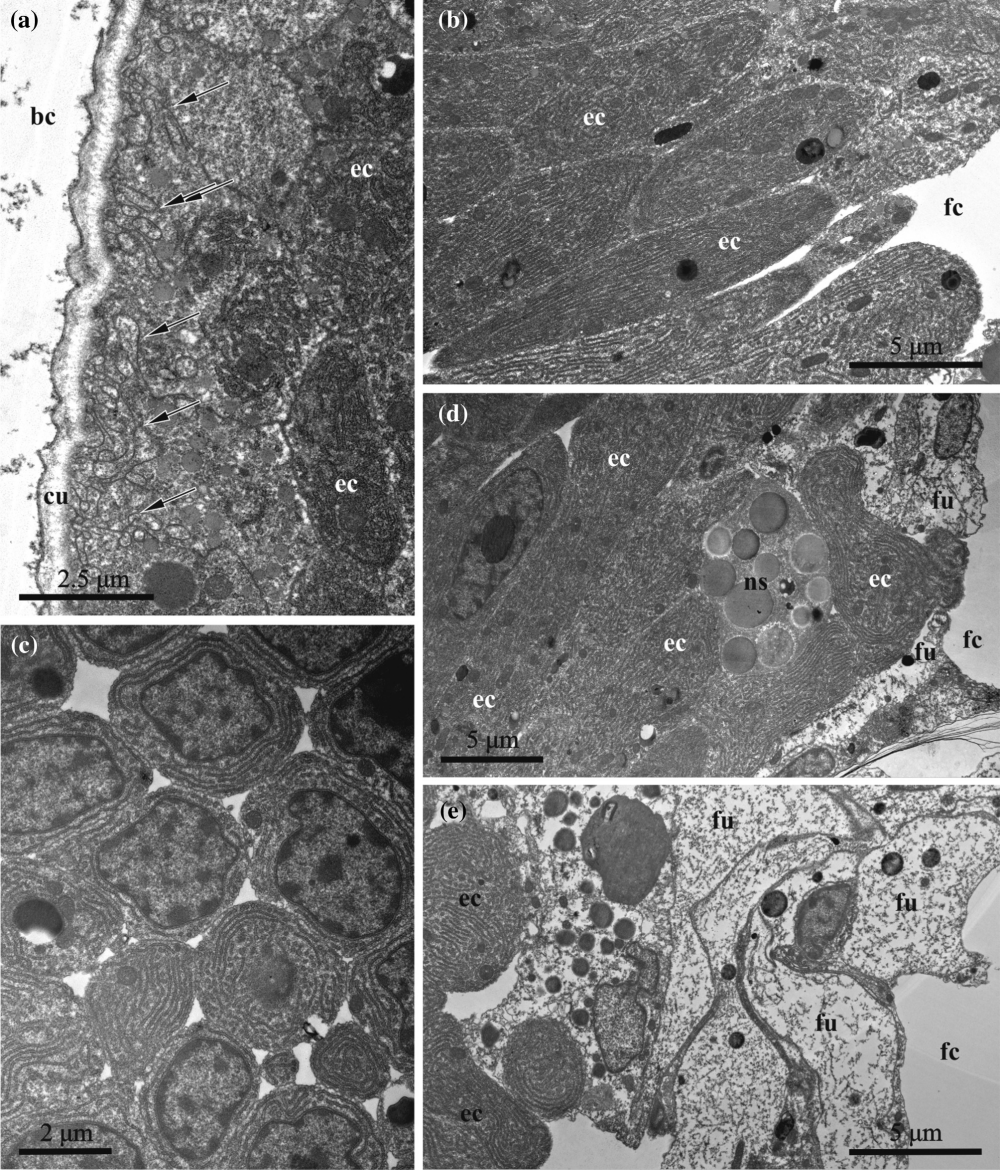
*C. hyalina,* embryophore at the middle‐stage of embryonic incubation (TEM). (a) Embryophore (nutritive) cells with numerous dense infoldings (indicated by arrows) underneath the cuticle and well‐developed RER in the basal part. (b) Longitudinal section of fusiform embryophore cells containing strongly‐developed RER; basal parts of the cell face the maternal coelom. (c) Cross‐section of the nutritive cells; section has been made through the tips of the cells in the basal part of embryophore that explains a presence of intercellular spaces. (d) Basal part of embryophore composed of the nutritive cells interspersed by the processes of the funicular cells; nutrient‐storage cell is visible between the nutritive cells. (e) Basal area of embryophore covered by the funicular cells with electron‐lucent cytoplasm and abundant inclusions. Abbreviations: bc = brood cavity; cu = cuticle of the distal zooidal wall; ec = embryophore (nutritive) cells; fc = coelom of female zooid; fu = funicular cells; ns = nutrient‐storage cell

**Figure 10:**
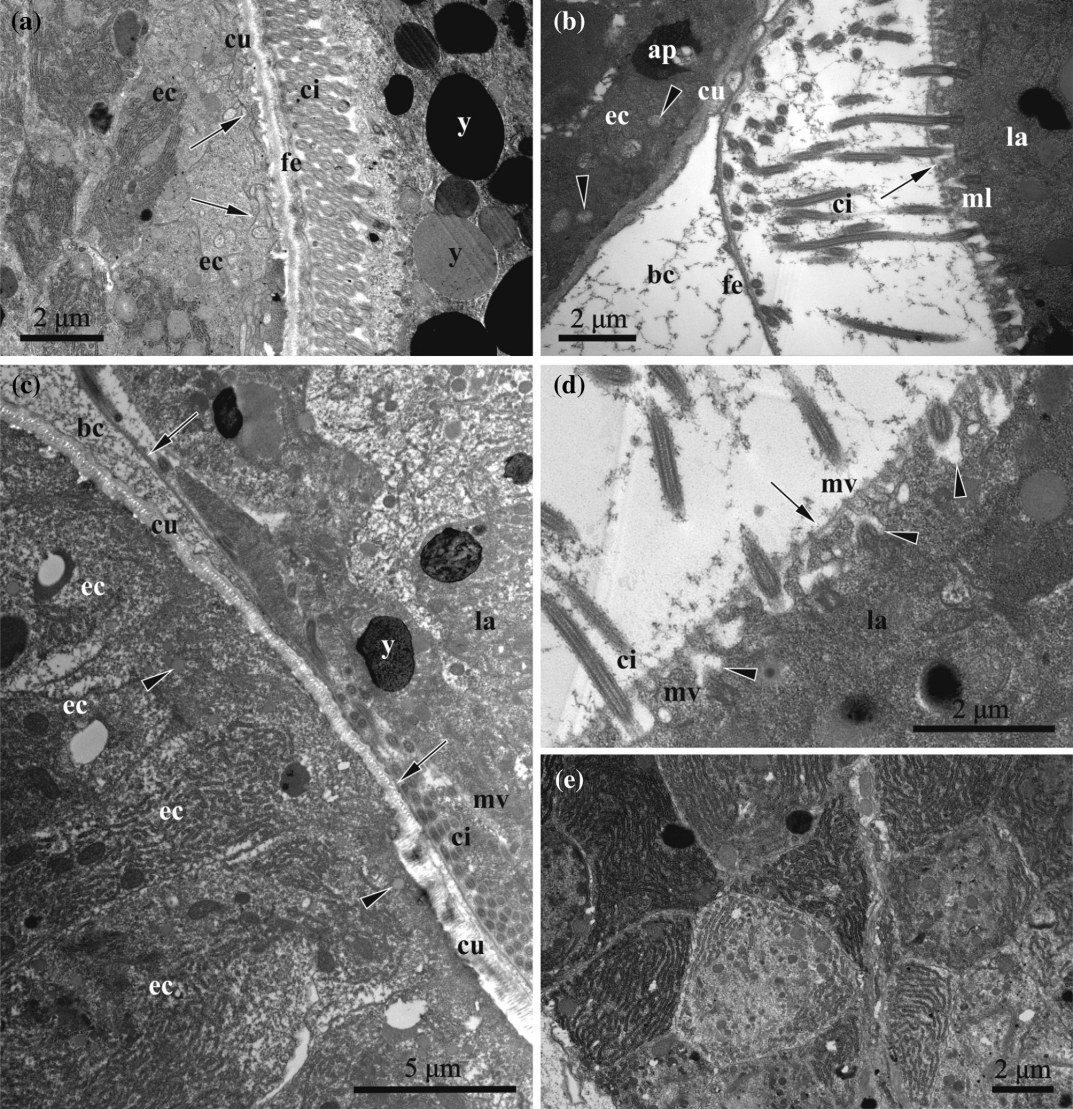
*C. hyalina,* embryophore at the middle (a) and advanced stage (b–e) of embryonic incubation (TEM). (a) Embryophore with the apical infoldings (indicated by arrows) underneath the cuticle (to the left) and early larva. (b) Advanced embryophore (to the left) devoid of infoldings and the early larva surrounded by fertilization envelope; vesicles (shown by arrowheads) with flocculent material are seen underneath the cuticle. Microvilli on the surface of the larval cells are embedded to glycocalyx (indicated by arrow). (c) Embryophore appressed to the advanced larva. Fertilization envelope shown by arrows, vesicles with homogenous material indicated by arrowheads. Larval cell in the upper part of the image has smooth plasmalemma, whereas the “lowermost” cell bears microvilli. (d) Close‐up of the larval surface showing endocytotic canals (arrowheads) between microvilli embedded into glycocalyx (shown by arrow). (e) Cross‐section of the embryophore cells with strongly developed RER. Flocculent material in the brood cavity is visible in (b), (c) and (d). Abbreviations: ap = autophagosome; bc = brood cavity; ci = larval ciliature; cu = cuticle of the distal zooidal wall; ec = embryophore (nutritive) cells; fe = fertilization envelope; la = larva; mv = microvilli; y = yolk granule

Embryo growth and development takes place in the ovicell. The zygote as well as the young embryo are noticeably smaller than the incubation cavity, being freely suspended in its fluid (Figures 2d and 5a). The embryophore is a temporary organ that begins developing directly after oviposition via multiplication and enlargement of the epidermal (and, possibly, peritoneal) cells of the non‐skeletal distal wall of the female zooid closing the ovicell opening. This wall has a cuticular sclerite for the muscle attachment in its upper part (Figure 5a,b), and is slightly swollen during embryonic incubation, thus being a reduced version of the ooecial vesicle known in most cheilostome brooders. The cytoplasm of the epidermal cells is intensively stained deep blue in histological sections (Figures 3d and 5). The cuticle of the non‐skeletal distal wall is thicker than that of the skeletal zooidal walls (up to 700 nm), but this does not prevent an exchange of substances between embryophore and embryo. The cuticle has a complex structure consisting of a thin electron‐dense peripheral layer composed of the parallel fibrils underlain by a much thicker striated layer represented by dense, slightly curved fibrils mainly perpendicular to the wall surface. Moreover, this layer is stratified, showing zones with different electron density, from the upper light to the middle dark and lower gray (Figures 8a,c‐e, 9a and 10c).

Fully formed larvae are released from the ovicell by contraction of the special muscles which wrinkle the distal zooidal wall that plugs the entrance to the brood cavity. Proximally, these paired muscular bands are attached to the skeletal basal wall of the female zooid by hemidesmosome‐like contacts (Figure 8c). Distally they are attached to the cuticular sclerite and to the cuticle of the distal wall between nutritive cells of the embryophore. While the opercular muscles (Figure 3d,e) are two large muscle bands attached to the opercular sclerite, the muscles of the distal zooidal wall (Figures 7b and 8a,b) are more complex: they are represented by two groups of bundles attached to the wall in its upper and lower part.

#### 3.4.1 Early developmental stage

Brooded embryos and larvae are surrounded by a thick fertilization envelope (Figures 5a, 8e, and 10b). Initially it adjoins the blastomeres of the early embryo, further retreating from them and leaving a substantial space between the envelope and the embryo/larva. The fertilization envelope consists of a thinner, electron‐dense external and a thicker, loose internal (lower) layer.

In the early embryo, the peripheral blastomeres have a slightly convoluted plasmalemma and show no signs of endocytosis. Instead, their cytoplasm is filled with large and numerous yolk granules (Figure 7e).

Oviposition and the onset of embryogenesis coincide with the development of the placental analogue in the distal wall of the maternal zooid, whose cells start to grow and divide (Figure 5a). In this process, the initial epithelial lining consisting of cuboidal and prismatic cells transforms to the early embryophore composed of elongated and irregularly shaped cells with overlapping basal (facing the coelom) parts and numerous intercellular spaces (Figure 8a, b). The cells of the embryophore, some interspersed with the muscles of the zooidal wall, are interconnected by elongated processes constituting a ‘loose layer’. In addition, embryophore cells multiply in the lower (close to the basal zooidal wall) part, forming a compact cell group below the distal wall (Figure 8c). Later, this cell group becomes indistinguishable, constituting a basal part of the embryophore, but it becomes well‐recognisable again after embryophore reduction (Figure 3e).

**Figure 11:**
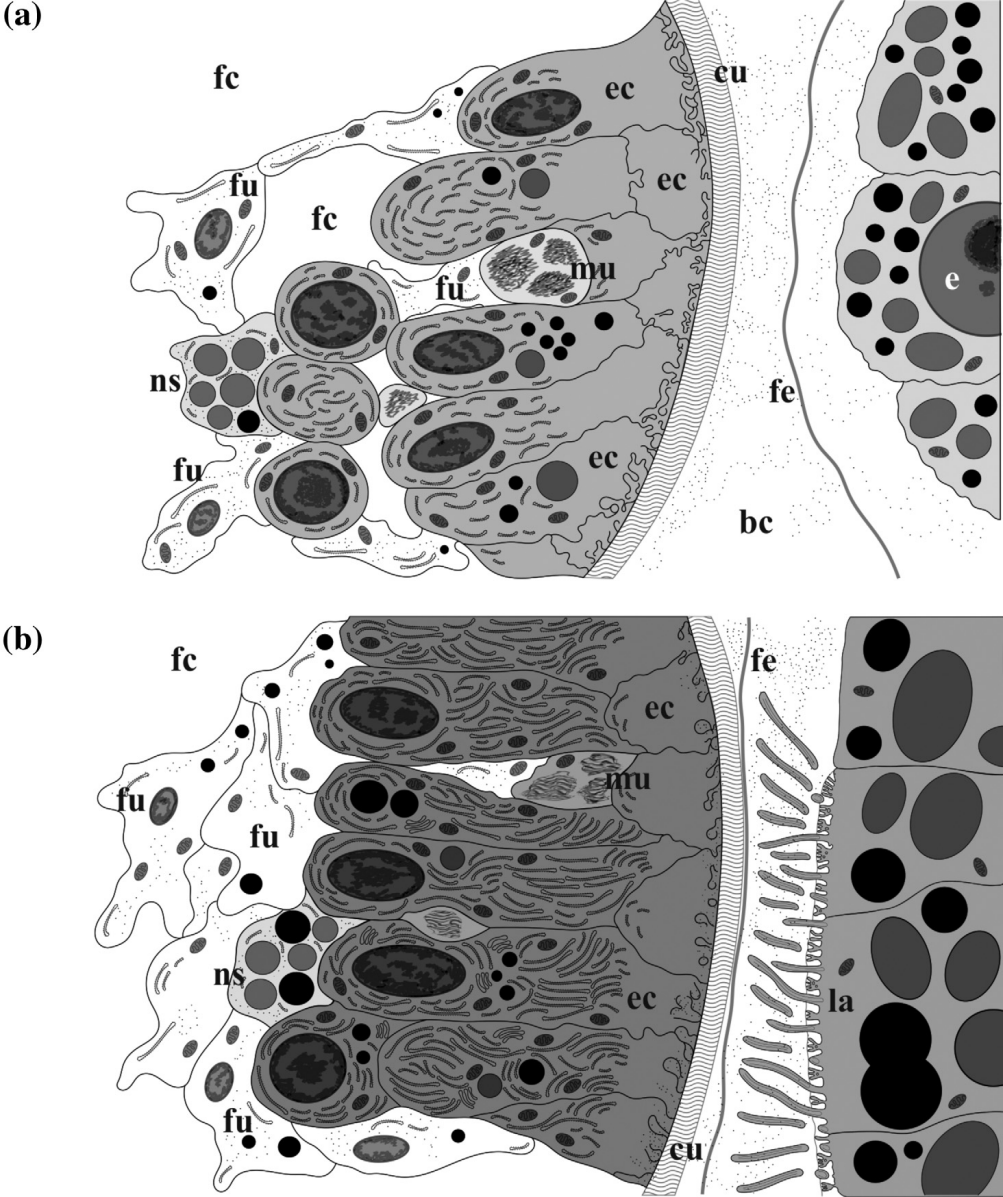
*C. hyalina,* scheme of the placental analogue and embryo on the early (a) and advanced (b) developmental stages. (a) Early embryophore (to the left) with numerous infoldings of the nutrient cells and the early embryo surrounded by fertilization envelope. (b) Advanced embryophore (to the left) with reduced number of infoldings and the early larva surrounded by fertilization envelope; ciliated larval cells form microvilli and endocytotic canals between them. Abbreviations: bc = brood cavity; cu = cuticle of the distal zooidal wall; e = embryo; ec = embryophore (nutritive) cells; fc = coelom of female zooid; fe = fertilization envelope; fu = funicular cells; la = larva; mu = muscle bundles of the distal zooidal wall; ns = nutrient‐storage cell

The ultrastructure of the embryophore cells (termed here and elsewhere as nutritive) is rather uniform. The cytoplasm is electron‐dense (gray). The large oval nucleus normally occupies most of the cell volume, exhibiting a big round nucleolus and heterochromatin predominantly aggregating on the nucleus periphery (Figure 8b). The cytoplasm contains numerous mitochondria and free ribosomes, Golgi apparatus and cisternae of RER. A few large yolk granules (both lipid droplets and protein platelets) and numerous round, smaller inclusions are visible in some of the nutritive cells (Figure 8). At that time, the apical parts of some nutritive cells begin to form deep infoldings underneath the cuticle, pointing to the start of exocytosis (Figures 8a,c–e and 11a). These infoldings are larger and more numerous in the adjacent areas of the neighbour cells. Putative nutrients accumulate on the outer surface of the embryophore cuticle as a thin dark layer of the flocculent material that also spreads into incubation cavity (Figure 8a,d,e).

Some irregularly shaped and sometimes folded cells of the funicular cords (hereafter termed funicular cells) with electron‐translucent cytoplasm and lobate nucleus are seen in the proximal part of placental analogue (Figures 8b and 11a). Nutrient‐storage cells are visible among the nutritive and funicular cells in the proximal part of the embryophore.

#### 3.4.2 Mid‐developmental stage

When the peripheral embryonic cells begin to develop cilia, the embryophore cells simultaneously greatly increase in size and number; most of them become trapezoid or fusiform and are oriented perpendicular to the distal zooidal wall (Figures 3d and 5b). Cell layers or groups are not recognizable. Instead, the placental analogue is a massive and complex nutritive organ composed of tightly‐packed, large cells (Figure 9a–c). Not all of them seem to contact the cuticle of the distal zooidal wall. The muscular bands of the distal wall are embedded in the embryophore.

During growth, the electron density of the cytoplasm of the nutritive cells increases, as does the number of mitochondria and various inclusions, that is, large, round or oval electron‐dense granules and smaller vesicles with grayish content. Nutritive cells show a strongly developed synthetic machinery including multiple free ribosomes and numerous cisternae of RER that become longer and more regularly arranged (often stacked). Noteworthy, such cisternae are predominantly situated basally in those nutritive cells that are adjacent to the cuticle of the embryophore (Figures 9a and 10a). In others, apart of the nucleus, the cisternae fill most of available cytoplasm (Figure 9a–d). In large, spherical or oval nuclei, a considerable proportion of the heterochromatin is scattered throughout the nucleoplasm.

The entire apical surface of the nutritive cells adjacent to the cuticle is covered with infoldings that become conspicuously deeper and complex. Flocculent material is seen in the brood cavity (Figure 8a).

Intercellular spaces between the nutritive cells remain only in the periphery of the embryophore (Figure 9b–e). Large funicular cells are met in the proximal part of placental analogue. They contact the fusiform cells and form a distinct part of the embryophore. Only few organelles are present. Small lipid droplets are accompanied by larger round or irregularly shaped protein inclusions. Funicular cells do not form a continuous layer, but are situated individually or in groups of two to several cells. Accordingly, some areas in the proximal part of embryophore are devoid of these cells. Nutrient‐storage cells are visible in the basal part of embryophore too (Figure 9d).

#### 3.4.3 Advanced stage

At the time when the embryo develops a ciliary corona, the placental analogue can occupy up to half(!) of the female zooid volume (Figure 5c). The embryophore is composed mostly of tightly pressed, elongated fusiform nutritive cells oriented along the zooid's longitudinal axis (Figures 10c,e and 11b). Their cytoplasm is electron‐dense. The nucleus is large, spherical or elongated, and contains large round nucleoli and heterochromatin scattered throughout the nucleoplasm. These cells are characterized by an extremely large amount of free ribosomes and densely packed cisternae of RER. Numerous mitochondria and multiple Golgi apparatuses are present. The cytoplasm contains spherical lipid droplets and protein platelets of different size, along with multivesicular‐like bodies and various inclusions that are sometimes aggregated in groups. Autophagosomes were rarely observed (Figure 10b).

Numerous funicular cells with electron‐translucent cytoplasm constitute the proximal part of the placental analogue, demarcating it from the zooidal coelomic cavity. They are irregularly shaped, often with processes, and contain large nucleus. Some of them are interspaced with fusiform nutritive cells. Funicular cells vary in the electron density of their cytoplasm, ranging from almost white to gray, and contain organelles and inclusions of the different size, shape and content: large autophagosomes, smaller vesicles with flocculent material, spherical lipid droplets and protein platelets. As opposed to fusiform nutritive cells, they contain less RER and fewer free ribosomes. Mitochondria and Golgi complexes, however, can be numerous (Figure 9e).

The fully developed placenta continuously releases flocculent material through the cuticle of the distal zooidal wall into the incubation cavity (visible on either side of the fertilization envelope of the incubated larva) (Figure 10b). No clear zonality of the cuticle is evident in many cases, but its lower margin often becomes convoluted. Infoldings of the apical parts of nutritive cells are reduced, ill‐defined or totally missing at this stage. Instead, numerous round and oval vesicles of various sizes, filled with either homogeneous or flocculent material, appear below the cuticle, and some of these vesicles seem to fuse with the plasmalemma (Figure 10b,c). Autophagosomes were recorded in this cell zone too.

The late embryo/early larva occupies the incubation cavity entirely and abuts the placental analogue (Figures 5c, 10a,c and 11b). Moreover, the pressure of the growing larva on the embryophore often causes it to curve towards the maternal zooid. Most surface cells on all sides of the larva—irrespective of which side faces the placenta—bear cilia and short microvilli. Tips of microvilli are embedded into the dense glycocalyx (Figure 10b). Numerous pinocytotic invaginations and canals as well as small vacuoles are clearly visible between the bases of the microvilli (Figures 10d and 11b). Some of the surface cells, however, have a smooth plasmalemma (without cilia or microvilli) and are not covered by a glycocalyx. Also, no endocytotic activity was recorded in areas with a smooth plasmalemma (Figure 10c and 11b). Embryos increase approximately 9‐fold during incubation in the ovicell, resulting in short‐lived endotrophic larvae (diameter 123.0–150.0 × 88.0–128.0 μm).

#### 3.4.4 Embryophore after incubation

**Figure 12:**
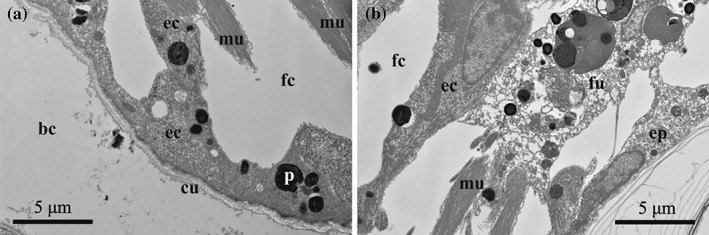
*C. hyalina,* embryophore after larval release (TEM). (a) Part of the embryophore, showing loose pattern of the cell placement with large intercellular spaces. (b) Basal part of the same embryophore with adjacent funicular cells. Almost no flocculent material is visible in the brood cavity. Calcified skeleton is visible in the right lower corner. Abbreviations: bc = brood cavity; cu = cuticle of the distal zooidal wall; ec = epithelial cells of the distal zooidal wall; fc = coelom of female zooid; fu = funicular cells; mu = muscle bundles of the distal zooidal wall; p = protein(?) platelet

After larval release, the placental analogue collapses. Both, nutritive and funicular cells become smaller and fewer, and intercellular spaces appear and expand between them (Figures 3e and 12). Their nuclei remain large and active, but the cytoplasm becomes more electron‐light and the synthetic apparatus mostly degrades (Figure 12a). No Golgi apparatuses were recorded, the cisternae of the RER recede and form an irregular pattern. Most inclusions disappear, whereas autophagosomes are present in the cytoplasm. The structure of funicular cells changes only minimally, except they also contain autophagosomes (Figure 12b). This condition persists until the next zygote is transferred to the ovicell, at which time the concurrent development of the embryo and placental complex repeats.

## 4 DISCUSSION

### 4.1 Remarks on the life cycle and placental reproductive strategy

Although *C. hyalina* has been an object of the long‐term experimental work (see above), many important details of its life‐history remained unknown or questionable. This also includes the number of generations per year and colony life span. Eggleston (1972), in his study on bryozoan life‐cycles and reproductive patterns in the Irish Sea, classified *C. hyalina* as a short‐lived species with colonies living less than 1 year. During a year, he recorded three periods with reproduction peaks (February–March, May–August and October–November). He interpreted these to indicate three separate generations, in which each colony produces embryos for a few weeks and dies soon after larval release. This view was corrected by Cancino (1986), who found that the maximum average life expectancy varied from 20 days to about 6 months in *C. hyalina* being constrained by the time and site of larval settlement on the kelp blades and the seasonal deterioration of the latter. Else, competitive overgrowth of the colonies by other epiphytes sometimes played a role. Based on his plots, the maximum life span of this species is 7 months. Interestingly, Hughes (2005), based on Cancino's data, gives a maximum life span of 9 months, obviously relying on the maximum life span of the kelps.

In contrast, some colonies from the White Sea population potentially could live at least 1 year (up to 15 months, from June of the preceding year to September of the current year) on kelps, depending on the time of establishment and successful survival during overwintering. Breakage of the substrate is a limiting factor for these colonies, but they can potentially live 1–2 months longer on red algae. Our estimations fit well to the experimental data: colonies of *C. hyalina* were maintained up to 18 months with repeated reproduction cycles on artificial substrata in the Irish Sea (Cancino & Hughes, 1987). Data on its life‐span on, for example, stones or shells, are absent, however.

Thus, although *C. hyalina* often dominates on ephemeral substrates (Cancino, 1986), at least some colonies living on algae are not ephemeral. Also, our observations, rather than showing a succession, revealed the co‐existence of at least three (but more likely four) generations in the White Sea, a situation that presumably exists in the Irish Sea as well. We should add here that a number of genetic studies demonstrated that *C. hyalina* is a complex of cryptic species (Gómez, Wright, et al., 2007; Gómez, Hughes, Wright, Carvalho, & Lunt, 2007; Hoare, Goldson, Giannasi, & Hughes, 2001; Hughes et al., 2008; Waeschenbach, Porter, & Hughes, 2012). Differences in the sexual performance of distant populations could help to recognize potential sibling species.

Eggleston (1972) noted that the life‐cycle of epibiotic species must be adapted to that of their living substrate. In those *C. hyalina* colonies that inhabit ephemeral substrates, the ability for early maturation is apparently such an adaptation (Cancino & Hughes, 1987; Hughes, 1989). Indeed, we found the colonies consisting of only 10 autozooids and 1–4 female polymorphs in June–July and, sometimes, in August. Such a very early start of reproduction is also known in other bryozoans, both brooders and broadcasters, living on algae (Bernstein & Jung, 1979; Yoshioka, 1982; Nekliudova, unpubl. data). Elsewhere, early sexual maturation is characteristic for interstitial bryozoans (Håkansson & Winston, 1985; Winston & Håkansson, 1986), suggesting that life in unpredictable conditions generally promotes early larval production. That strategy can be viewed not only in connection with potential risks (e.g., substrate destruction, Hughes, 1989), but also under favourable conditions when abundant food allows allocating energy to reproduction soon after colony establishment (Nekliudova, unpubl. data).

Dyrynda and Ryland (1982) were the first to report that placentation is characteristic for species with ephemeral colony parts. They suggested (although incorrectly argued, see Ostrovsky, 2013a) that placentation could provide faster larval production, enabling more offspring to be released in a shorter time. Ostrovsky supported, but transformed, this idea, arguing that placental brooders combine shorter oogenesis with simultaneous embryonic growth and development during incubation (Ostrovsky, 2013a; Ostrovsky et al., 2009, 2016). For example, the entire reproductive cycle, from oocyte formation to larval release, takes 4 weeks in *Callopora dumerilii* (Silén, 1945) and 6 weeks in *Chartella papyracea* (both non‐placental brooding cheilostomes; Dyrynda & King, 1983). In contrast, this period was only 3 weeks in the matrotrophic *Bugulina flabellata* (Dyrynda & King, 1983; Dyrynda & Ryland, 1982) and *B. simplex* (Grave, 1930; Ryland, 1974) (both as *Bugula*). Such faster reproduction could be especially effective in ‘seasonal’ seas, enable faster occupation of vacant niches after, for example, overwintering. In *C. hyalina* Hughes (1987) observed that the duration of one reproductive cycle was 14–17 days (comparable with the mentioned placental bugulids). In contrast, Cancino and Hughes (1988) reported 3–4 weeks for embryonic development alone, and this difference could be explained by seasonality in the Irish Sea.

Because the large part of the studied populations of *C. hyalina* is represented by short‐living colonies, this species potentially could use the advantages of placental strategy. Its colonies inhabit a large spectrum of substrates, both stable and ephemeral, organic and not (Gostilovskaya, 1978; Hayward & Ryland, 1999; Kluge, 1975). Considering this, we speculate that rapid larval production supported by placentation is an important factor explaining the success of this species in boreal and Arctic seas.

### 4.2 Fertilization

In male polymorphic zooid, the polypide consists of a functional lophophore without a digestive tract. There is also a system of organs (polypide retractor muscle, the occlusor muscles of the operculum and parietal muscles that expand the large hydrostatic sac (ascus) (Hughes, 1987; descriptions by Marcus (1938) do not belong to *C. hyalina*) that serves for the tentacle protrusion followed by the sperm release (Cancino & Hughes, 1988; Hoare et al., 1999; Manríquez et al., 2001). Due to the presence of a similar system of organs for polypide excursion in the female zooids, the female lophophore should be functional, enabling sperm capture and entry (as well as oviposition to the ovicell) (Ostrovsky, 1998; our data; but see Hughes, 1987). As in other cheilostomes, sperm presumably enters the female coelom via the supraneural coelomopore and precociously fuse with the early (previtellogenetic) ovarian oocyte (Bishop et al., 2000; Ostrovsky, 2013a; Ostrovsky & Porter, 2011; Temkin, 1996; our data).

Spermatozoa between two follicle cells in the ovary of *C. hyalina* were first reported by Hughes (1987). Later, they were found in the ovaries between the follicle cells as well as in the previtellogenic and vitellogenic oocytes (Ostrovsky, 1998, 2013a). In all these cases, the logical route of sperm toward the ovary is through the coelomic cavity. In this respect, our finding of sperm between funicular cells near the ovary is of interest. Could sperm also use funicular cords for this purpose?

In *Celleporella* sp. (as *C. hyalina*), Marcus (1938) found sperm in all three zooidal types, including autozooids. Elsewhere, it was recorded in the cavity of the ooecium (protective capsule of the ovicell) and in the coelom of an incipient female polymorph that had no vestibule yet (Ostrovsky, 1998). Moreover, experiments showed that alien sperm could be stored by small colonies (three autozooids) for several weeks and used only when the female polymorphs develop (Hughes, Manriquez, & Bishop, 2002). Therefore, in all these cases, the sperm, once caught, somehow travel through the colony. Marcus (1938) suggested that communication pores were the pathway, but this was questioned by Hughes (1987) because of the presence of the pore‐cell complexes plugging these pores (see also Ostrovsky, 1998, 2008; Reed, 1991). In contrast, Hughes et al. (2002a) speculated that the funicular strands could be used for sperm translocation, not considering the fact that these strands are interrupted by the pore‐cell complexes. Finally, Ostrovsky (2013a) suggested migration via budding sites prior to the completion of transverse walls (and, thus, communication pores and their cell plugs) between autozooids and female polymorphs.

The current finding, which seemingly supports the suggestion of Hughes et al. (2002), is puzzling. Although the central lumen is present in the cheilostome funicular cords (Carle & Ruppert, 1983), no data are available to indicate that the sperm move from this lumen inside the pore‐cell complex, further squeezing between its cells, and thus traveling to the neighbour zooid (see Mukai, Terakado, & Reed, 1997 for discussion). If, however, that is possible, then those cords that lead from the pore to the ovary are a potential route for the sperm. Another possible explanation is that these sperm in trying to reach the ovarian oocytes inadvertently entered the funicular cords adjacent to the ovary.

### 4.3 Oogenesis and mechanisms of yolk synthesis

Studies on invertebrate oogenesis generally consider major traits such as the origin of the primordial germ cells and the mode of oogenesis, including mechanisms of yolk synthesis (Aisenstadt, 1984; Raven, 1961; Wourms, 1987). Reed (1991) suggested that epigenetic germ cell formation is characteristic for many colonial invertebrates including bryozoans. This reflects the ability of somatic cells to dedifferentiate into totipotential cells transforming to the primordial germ cells and, thus, possibility of germ cell determination throughout ontogeny. Extavour and Akam (2003) also concluded epigenesis to be the basal mode of germ cell specification in Metazoa, including lophophorates, in which primordial germ cells develop in/from either mesenchyme or peritoneal epithelium during late embryogenesis or post‐embryogenesis. In Bryozoa, female germ cells appear within the mesothelial lining of the forming polypide bud or zooidal wall. Cells of the mesothelial lining surrounding the germ cells form the follicle wall around growing oocytic doublets (reviewed in Ostrovsky, 2013a). This view is consistent with the finding of the presumed oogonial doublet associated with developing female polypide (Ostrovsky, 1998, 2013a), and with our data on the early ovaria in *C. hyalina*.

Although only a few oocytes ultimately develop into the larvae by a single female zooid, the ovarian germ cells can be numerous (up to 25). This points to excessive oogonia/oocyte production in the studied bryozoan. Such a ‘surplus’, together with the resorption of some germ cells in the ovary, could be an ancestral condition known in broadcasting species (Hageman, 1983), but has never been reported in the placental cheilostomes that normally produce limited number of the germ cells (Ostrovsky, 2013a).

Three modes of metazoan oogenesis can be distinguished regarding the accessory cells: solitary (oocytes develop without such cells), nutrimentary (oocyte is supported by the special nurse cell[s] of either germ or somatic origin), and follicular (each oocyte develops in a follicle, formed by somatic cells, performing either supportive or nutritive function, or both; Aisenstadt, 1984; Wourms, 1987). Most of the brooding cheilostomes combine nutrimentary and follicular modes, although the nutritive role of the follicle cells has been studied ultrastructurally in just three species (Dyrynda & King, 1983; Moosbrugger et al., 2012). Yet, not all bryozoan brooders possess nurse cells (Ostrovsky, 2013a).

The presence of an intercellular bridge connecting the oocyte and its nurse cell suggests an intimate physiological connection between the siblings in *C. hyalina*. Hughes (1987) proposed that the nurse cell is a nutrient source for the oocyte during early vitellogenesis. In our opinion, such relationships exist during the entire period of yolk accumulation. Several lines of evidence point to the high absorbing and synthetic activities of this sibling that can send yolk precursors, RNA and ribosomes to the oocyte (discussed also in Wourms, 1987). These include the development of microvilli, signs of yolk synthesis, a large active nucleus in the nurse cell (which grows much faster than the cell itself), and numerous free ribosomes in its cytoplasm. The same relationships between the oocyte and its nurse cell were suggested in the cheilostome brooders *Chartella papyracea*, *Bugulina flabellata* and *Bicellariella ciliata* (Dyrynda & King, 1983; Moosbrugger et al., 2012). It should be stressed that the complex nature of the intercellular bridge, consisting of cytoplasmic and membraneous areas with tight junctions in *C. hyalina* has been described for the first time in bryozoans.

The follicular cells enveloping the vitellogenetic doublet actively participate in vitellogenesis, using their strongly developed synthetic apparatus, that is, numerous RER cisternae and free ribosomes, mitochondria as well as Golgi complexes. The clathrin‐coated pits in the oolemma, suggest that follicular cells synthesize and release nutrients absorbed by the growing oocyte. Note that the electron density of the cytoplasm differs in different follicle cells, suggesting their different functions (e.g., specialization in the synthesis of different products). Consequently, oogenesis in *C. hyalina* is a combination of nutrimentary and follicular types, as in most incubating cheilostomes (Dyrynda & King, 1983; Moosbrugger et al., 2012). This contrasts to non‐brooding and, at least, one brooding species, in which oogenesis is exclusively of the follicular type (Hageman, 1983; Reed, 1991; Shevchenko, unpubl. data).

The mechanism of vitellogenesis depends on the type of yolk precursors obtained by the developing oocyte, and can be autosynthetic, heterosynthetic (Schechtman, 1955) or mixed (Eckelbarger, 1983). The development of the microvilli and massive synthetic apparatus (large active nucleus with convoluted membrane, RER cisternae as well as multiple mitochondria and Golgi apparatus) in both cells of the vitellogenic doublet indicate active transport of low weight molecular precursors and autosynthetic vitellogenesis in them (Eckelbarger, 1994). Even though the microvilli and ribosomes are still numerous, the Golgi complexes and RER cisternae become less prominent in the final stages of vitellogenesis, indicating a decrease in autosynthetic activity. At the same time, the strongly developed synthetic apparatus in the surrounding follicle cells and the presence of the coated pits in the oolemma of the oocyte point to heterosynthesis. Thus, the vitellogenesis mechanism is mixed in *C. hyalina*, like in four previously studied cheilostomes (Dyrynda & King, 1983; Hageman, 1983; Moosbrugger et al., 2012).

Interestingly, we detected neither ‘direct bathing’ of vitellogenic oocyte in the coelomic fluid, nor ‘nutrient‐storage cells’ in the zooidal peritoneal layer opposite to the apical pole of the mature oocyte as described by Hughes (1987, p. 703). In his Plate VII(a) the oocyte is shown to be covered by a thin yet prominent follicular layer. The ‘nutrient‐storage cells’ filling the distal half of the female zooid—judging from their location, description and the photographs provided—are probably nutritive cells of the embryophore at the early stages of its formation or, possibly, cells of the rudimentary polypide. The cells with numerous large inclusions reported in our study (that we also termed ‘nutrient‐storage cells’) are more similar to the cells described by Dyrynda and King (1983) in zooids of *Bugulina flabellata*. In both cases, these cells were associated with the peritoneum of the cystid wall, funicular cords or gonads and contained large spherical yolk‐like inclusions.

The increase in oocyte volume during vitellogenesis estimated in the present study (37.5 times) exceeds the calculations made by Ostrovsky (1998) by almost three times, which we explained by the absence of late oocytes in his material. Accordingly, the oocytes were incorrectly described as microlecithal in *C. hyalina* (Ostrovsky, 1998), although they are in fact macrolecithal (Ostrovsky, 2013a, 2013b; our results).

Dyrynda and King (1983) described a fibrous ‘primary coat’ as a precursor of the ‘vitelline envelope’ surrounding ovarian vitellogenic oocytes in *C. papyracea* and *B. flabellata*. According to their description, the oocyte microvilli are embedded in this coat. In contrast, this structure was not recognized around ovarian oocytes in *B. ciliata*, although the fertilization envelope surrounding the brooded embryo is easily recognizable in this species (Moosbrugger et al., 2012). Instead, the oocyte microvilli were described as being embedded in a thick matrix of medium electron‐density that is actually very similar to the ‘coat’ described in the two aforementioned species and the fertilization membrane in *C. hyalina*. Based on this similarity, we suggest that the above mentioned matrix is a fertilization membrane in *B. ciliata*, permeable for both low and high weight molecular products delivered by the follicle cells.

### 4.4 Development and functioning of the placental analogue

In matrotrophic cheilostomes, every brooding episode is accompanied by temporal hypertrophy of the embryophore, which collapses after larval release. Ostrovsky (2013a) recently suggested that the embryo produces signal molecules stimulating placental analogue formation and functioning because the embryophore develops soon after oviposition and ceases synthetic activity and degenerates directly after larval release. In *C. hyalina*, nutritive cells of the placental analogue seem to be its main synthetic part. This is based on the fact that they are much larger and more numerous, and that synthetic organelles develop extensively during embryogenesis. Judging from their position, nutritive (mostly, fusiform) cells could partly originate from the epithelial lining of the distal zooidal body wall and partly from the peritoneum (although it is very loose in gymnolaemate bryozoans, see Mukai et al., 1997). Currently, in *C. hyalina* we are unable to distinguish the cells of possibly different origin because they are neither organized in prominent layers nor display any distinction in structure.

In the studied species during early embryonic development, the energy costs are apparently covered by the yolk of the egg. Soon thereafter, the developing embryophore starts secreting nutrients into the incubation cavity seen as electron‐dense flocculent material. No pores or channels in the cuticle were recorded, which suggests that the nutritive material passes through it in a soluble state (also suggested by Hughes, 1987). As postulated for *Bugula neritina* and *Bicellariella ciliata*, diffusion and the osmotic gradient can be the driving forces moving the dissolved nutritive matter across the cuticle (Moosbrugger et al., 2012; Woollacott & Zimmer, 1975). During the period of active nourishment, the fusiform cells make up most of the placental complex and contain a strongly developed synthetic apparatus (see above). The infoldings formed by the apical membranes of nutritive cells at the early and middle stages of embryo incubation are probably a sign of nutrient secretion. This reflects either a surface increase for transmembrane transport or active exocytosis. These infoldings correspond to similar structures formed by nutritive cells of the bugulid placental cheilostomes (Moosbrugger et al., 2012; Woollacott & Zimmer, 1975). Nonetheless, the extensive arrays of foldings developed by embryophore cells in the studied bugulids during the active phase of placentation are not characteristic for *C. hyalina*. In the latter, the infoldings are developed not so strongly, and present mainly during the early and middle incubation phase. Vesicles filled with flocculent or homogenous material that replace infoldings of the nutritive cells indicate a shift to another mechanism of exocytosis at the advanced stage.

During, active phase of nourishment, the funicular cells that are the part of the embryophore also increase in number and rearrange. They form a plexus in the basal part of the placental analogue with cytoplasmic processes passing between fusiform cells. Presence of the nutrient‐storage cells could also point to accumulation of nutrients in this zone. Contact of the funicular cords with interzooidal pore‐cell complexes indicates their main function as pathways for nutrient transport from the neighbouring feeding zooids. At the advanced stages of embryonic development, numerous mitochondria, Golgi complexes and inclusions with different contents, are present in the funicular cells. This may reflect intensified transport activity. An increased contact of the funicular plexus with the basal surface of the hypertrophied placental epithelium has also been detected in *Bugula neritina* (Woollacott & Zimmer, 1975).

The maximal enlargement (9‐fold) of the embryo during the brooding period in our material was less than Hughes (1987) estimated for the Irish Sea (15‐fold) but conforms to the data Ostrovsky (2013a) presented (8.8‐fold) for the White Sea. Such variation is not surprising and is known within and between populations in the placental cheilostomes (Marshall & Keough, 2003; Ostrovsky, 2013a, 2013b). As the oocyte size was the same in all these studies (about 80 μm), placentation apparently determines larval size. We predict that such variability should be common in all matrotrophic bryozoans.

### 4.5 Ovary versus placenta

Based on histological sections, Ostrovsky (2013a) recognized that the structure of the mature ovary differs in cheilostomes with different reproductive patterns. In contrast to non‐matotrophic brooders, placental species have far fewer follicle cells and usually no prominent subovarian zone that presumably plays an important role in the oocyte nourishment. This reflects a lower investment in progeny during oogenesis. For example, the follicular wall of the ovary in the placental *Bugulina flabellata* consists of a single layer of squamous cells with small cone of columnar cells at one pole. No subovarian space was detected in this species by both TEM and light microscopy (Dyrynda & King, 1983; Ostrovsky, 2013a). The same structure was documented in the con‐familiar *B. neritina* (Mathew, Schwaha, Ostrovsky, & Lopanik, 2018; Ostrovsky, 2013a). In contrast, in the non‐placental brooder *Chartella papyracea* the follicle wall consists of two cell layers, the lower of squamous and the upper (external) of columnar cells. Moreover, the coverage of the vitellogenic doublet by columnar cells is greater, reflecting the greater demand for yolk in oocyte production. The situation is similar in most non‐matrotrophic brooders studied that also possess a prominent ‘subovarian’ or ‘intraovarian’ space/zone filled by so‐called ‘basal’ cells and numerous intracellular spaces between them (Ostrovsky, 2013a).

Noteworthy, the ovarial structure of *C. hyalina* is reminiscent of both aforementioned variants. The basal part of its follicle on the mid‐vitellogenic stage includes cuboidal cells, thus resembling non‐placental cheilostomes. Conversely, the mature ovary consists of flattened and squamous cells, thus being more similar to the ovaries in most placental species. Also, although we did not find a sub‐ovarian space in the female gonad, we did detect intercellular spaces filled with electron‐dense material. Importantly, however, the sub‐ovarian zone has been recorded in some placental species although an additional ultrastructural study is required to confirm this issue (Moosbrugger et al., 2012; Ostrovsky, 2013a).

Despite the different contribution of the ovary to vitellogenesis in different species, the structure and function of the follicle cells are basically similar in all studied cheilostomes. Judging from their ultrastructure, they potentially obtain the low weight molecular precursors from the funicular cells contacting the follicle wall, although we found no intercellular contact between them. The coelomic fluid is another possible source. These precursors are clearly partially transported and partially modified intracellularly to more complex products. They are subsequently extruded into the intercellular spaces between follicular cells, as well as between them and the vitellogenic oocyte doublet. The presence of microvilli points that the latter absorbs this material via transmembrane transport added by endocytosis. The same processes obviously hold true for the embryophore, whose nutritive cells are also in contact with both the funicular cells and the coelomic fluid. Because intercellular junctions were not found, the nutritive cells presumably obtain low‐weight molecular nutrients by facilitated diffusion from the intercellular spaces between them and funicular cells as well as from the coelomic fluid directly. Further, nutritive cells transform and transport them to the embryo. Because of their similar functions, the follicular cells of the ovary and the nutritive cells of the embryophore, although of different origin, are ultrastructurally comparable.

Similar to the ovaries, the hypertrophy of the embryophore cells varies in placental cheilostomes. This is reflected in species with prominent, moderate and weakly developed placentas, which usually correlates with the degree of development and activity of the follicle epithelium in the ovary. Interestingly, the hypertrophy of embryophore cells does not always correlate with a degree of embryonic enlargement, and some species possess moderately developed, but functionally active embryophore (Ostrovsky, 2013a, 2013b).

The general structure and functioning of the placental analogues are similar among cheilostomes, involving multiplication and hypertrophy of the epithelial cells of the body wall lining surrounding the brood cavity, that is, either an internal brood sac or ovicell (Ostrovsky, 2013a). These processes are accompanied by the strong development of the synthetic machinery. Nonetheless, in almost all placental species the embryophore consists of one layer of hypertrophied nutritive cells and associated ‘sublayer’ of funicular cells. *C. hyalina* is an exception, having a more complex and massive nutritive organ that includes nutritive, funicular (transport) and nutrient‐storage cells. When fully developed it occupies a substantial part of the female zooid cavity. Only the placental analogue of *Costaticella solida* shows some similarity with this ‘nutritive tissue’, but its nutritive cells are less numerous and the funicular cells constitute about a half of embryophore (Ostrovsky, 2013a).

Strict polarity in organelle arrangement in hypertrophied cells of embryophore has been documented for the placental analogues of bugulids (Moosbrugger et al., 2012; Woollacott & Zimmer, 1975). This is also true for the nutritive cells adjacent to the cuticle in *C. hyalina*, whereas the rest (majority) of such cells do not show this polarity.

### 4.6 Embryonic nutrient uptake

Surface embryonic cells do not show signs of endocytosis until the stage when cilia are formed in the embryos of *C. hyalina*. Active transmembrane transport of the low weight molecular products is a probable mechanism of nutrient uptake at this early stage. Microvilli developing on the cell surface during late embryogenesis increase its absorption surface, pointing to active transmembrane transport too. Simultaneously, pinocytotic invaginations, channels and vesicles become visible near the microvilli bases. This makes pinocytosis a key mechanism of nutrient intake during larval development (see also Hughes, 1987).

As in *B. ciliata* (Moosbrugger et al., 2012), microvilli and pinocytotic canals are formed all over the embryo in *C. hyalina* (except for some cells devoid of cilia and microvilli), that is, not being restricted to the area adjacent to the embryophore. Accordingly, an uptake of nutritive material occurs around the entire embryonic surface. Importantly, in most placental cheilostomes studied so far, growing embryos are suspended in the much larger brood cavity during most of their development; they are not in contact with the embryophore (Moosbrugger et al., 2012; Ostrovsky, 2013a, 2013b). This makes histotrophy (absorbotrophy) a major nutritive mechanism during this period. The larva occupies the entire brooding cavity and abuts the embryophore only during the final stage of its development (questioned by Hughes, 1987). Thus, the placenta‐like system of the apposed embryo‐parent tissues providing physiological exchange (Mossman, 1937) ‘formally’ exists only during the last stage of incubation. Note here that it must provide bidirectional transport of substances, also removing wastes from the developing offspring.

Nutrient uptake in *B. neritina* reportedly occurs via a specialized region of the embryonic epithelium (presumptive internal sac tissue) directly opposed to the embryophore during most of embryogenesis. This specific area exhibits apical infoldings, pinocytotic channels and vesicles, pointing to its high absorptive capacity. In contrast, regions of the embryonic epithelia that do not abut the embryophore lack such infoldings (Woollacott & Zimmer, 1975). Published photos of histological sections of incubated zygotes and mid‐stage embryos confirm the early establishment of their contact with the embryophore in the related *Bugulina flabellata* (Ostrovsky, 2013b; Ostrovsky et al., 2009), although it is puzzling why zygotes should stick to the maternal wall. Nonetheless, we assume that absorption could also occur through the rest of the larval surface, although additional study is needed to resolve this question.

## 5 CONCLUSIONS

Matrotrophic nourishment encompasses numerous structural and physiological adaptations reflecting stages and trends in the evolution of parental care. Bryozoa, with their wide distribution of placental analogues that evolved multiple times within various clades, is a unique model showing various manifestations of matrotrophy from both parent and embryo. The placental analogue in *C. hyalina* strongly differs from all those previously described in matrotrophic cheilostomes. This suggests its independent origin within the family Hippothoidae, in which matrotrophy is known only in the genus *Celleporella* (Hughes, 1987; Marcus, 1938; Ostrovsky, 1998; Ryland, 1979). This is also confirmed by its position in the bryozoan molecular tree, where it clusters with non‐placental taxa (Taylor & Waeschenbach, 2015; Waeschenbach, Taylor, & Littlewood, 2012).

In matrotrophic Cheilostomata, the general similarity in the ultrastructure of the follicle and embryophore cells is not surprising because they all serve to transport and transform nutrients for the growing offspring. At the same time, both the follicle and embryophore cells show various (and correlated) degrees of development (i.e., cell size and number). This illustrates the consecutive stages in the shift from macro‐ to oligolecithal oogenesesis, accompanied by the placental advancement and transition from the incipient to substantial matrotrophy. Reduced follicle cell size and number, and the production of oligolecithal oocytes with a corresponding strong enlargement of the embryophore and, consequently, embryo, is clearly the most advanced variant of matrotrophic reproduction. It is known in only few species, however. Incipient matrotrophy with weakly developed placenta and macrolecithal oogenesis has been recently described in a few species too. Finally, a number of species, including *C. hyalina* show a mixture of traits, having macrolecithal, but relatively small, oocytes developing in the ovary formed by the flattened follicle cells, along with a modestly or strongly developed placenta providing a substantial embryo size increase (Moosbrugger et al., 2012; Ostrovsky, 2013a, 2013b; this study). This combination of traits has an intermediate position in the continuum of variation of matrotrophic provisioning describing non‐placental and placental cheilostome brooders.

## AUTHORS CONTRIBUTIONS

UN, TS, OK, DG and NC conducted all the practical work, and AO co‐ordinated research. UN and AO drafted the manuscript. All the authors read and approved the final version of the manuscript.

## Supporting information


**Table 1** Dates of sampling in 2012, 2013 and 2014, and the numbers of the studied colonies of *Celleporella hyalina*. The order of the dates is given in accord to the month sequence from May to September.
**Table 2.** State of the studied colonies of *Celleporella hyalina* (separately for overwintered and young age group). The order of the dates of sampling is given in accord to the month sequence from May to September. Empty boxes mean an absence of embryos in the colonies, hyphens indicate an absence of the colonies in the sample.
**Table 3.** Number of studied ovaries of *Celleporella hyalina* (collected in 2013 and 2015).
