# Supplementary material for: Sexual reproduction of the placental brooder Celleporella hyalina (Bryozoa, Cheilostomata) in the White Sea
Source: J Morphol. 2019 Jan 17;280(2):278–99. doi: 10.1002/jmor.20943 (PMC6949948; doi:10.1002/jmor.20943)
Supplement: Supplementary file 1 — Table 1 Dates of sampling in 2012, 2013 and 2014, and the numbers of the studied colonies of Celleporella hyalina. The order of the dates is given in accord to the month sequence from May to September. Table 2. State of the studied colonies of Celleporella hyalina (separately for overwintered and young age group). The order of the dates of sampling is given in accord to the month sequence from May to September. Empty boxes mean an absence of embryos in the colonies, hyphens indicate an absence of the colonies in the sample. Table 3. Number of studied ovaries of Celleporella hyalina (collected in 2013 and 2015). [file JMOR-280-278-s001.doc]

**Supplementary material**

**Table 1.** Dates of sampling in 2012, 2013 and 2014, and the numbers of the studied colonies of *Celleporella hyalina.* The order of the dates is given in accord to the month sequence from May to September.

| **Sampling date** | **Number**  **of overwintered colonies** | **Number**  **of young colonies** |
| --- | --- | --- |
| 31.05.2014 | 13 | 0 |
| 03.06.2014 | 41 | 0 |
| 07.06.2014 | 3 | 0 |
| 18.06.2014 | 21 | 1 |
| 19.06.2012 | 52 | 11 |
| 23.06.2012 | 96 | 3 |
| 27.06.2012 | 94 | 1 |
| 01.07.2012 | 18 | 1 |
| 15.07.2014 | 1 | 6 |
| 20.07.2014 | 1 | 8 |
| 24.07.2014 | 3 | 11 |
| 31.07.2013 | 0 | 15 |
| 06.08.2014 | 3 | 47 |
| 10.08.2014 | 3 | 92 |
| 15.08.2014 | 1 | 71 |
| 19.08.2014 | 0 | 159 |
| 23.08.2014 | 0 | 30 |
| 27.08.2014 | 0 | 104 |
| 07.09.2014 | 3 | 17 |
| 13.09.2014 | 0 | 2 |
| 17.09.2014 | 1 | 14 |
| 21.09.2014 | 0 | 25 |
| 25.09.2014 | 1 | 37 |
| 29.09.2014 | 0 | 8 |

**Table 2.** State of the studied colonies of *Celleporella hyalina* (separately for overwintered and young age group)*.* The order of the dates of sampling is given in accord to the month sequence from May to September. Empty boxes mean an absence of embryos in the colonies, hyphens indicate an absence of the colonies in the sample.

| **Sampling date** | **Overwintered colonies** | | | **Young colonies** | | |
| --- | --- | --- | --- | --- | --- | --- |
|  | **Mean colony size (mm²)**  **± standard error** | **Mean number of female zooids in a colony**  **± standard error** | **Presence**  **of embryos** | **Mean colony size (mm²)**  **± standard error** | **Mean number of female zooids in a colony**  **± standard error** | **Presence**  **of embryos** |
| 31.05.2014 | 30,13±4,63 | 46,00±19,44 |  | - | - | - |
| 03.06.2014 | 25,35±4,05 | 21,98±5,70 |  | - | - | - |
| 07.06.2014 | 26,33±1,79 | 0,00 |  | - | - | - |
| 18.06.2014 | 18,46±2,43 | 3,86±2,38 |  | 6,75±0,00 | 2,00±0,00 | + |
| 19.06.2012 | 7,41±0,79 | 1,25±1,01 |  | 2,20±0,55 | 0,27±0,27 |  |
| 23.06.2012 | 6,66±0,75 | 1,64±0,83 |  | 2,13±0,37 | 0,00 |  |
| 27.06.2012 | 9,42±0,92 | 9,30±3,03 |  | 5,50±0,00 | 0,00 |  |
| 01.07.2012 | 8,41±1,72 | 1,00±0,57 |  | 4,80±0,00 | 0,00 |  |
| 15.07.2014 | 14,20±0,00 | 0,00 |  | 5,60±0,86 | 0,00 |  |
| 20.07.2014 | 13,00±0,00 | 0,00 |  | 11,38±1,90 | 0,00 |  |
| 24.07.2014 | 22,03±1,77 | 9,00±4,51 | + | 14,32±3,70 | 9,55±9,55 | + |
| 31.07.2013 | - | - | - | 23,49±3,62 | 17,80±6,20 |  |
| 06.08.2014 | 37,71±8,86 | 195,33±31,35 | + | 3,37±0,42 | 0,00 |  |
| 10.08.2014 | 39,83±10,05 | 51,67±11,20 | + | 33,81±1,91 | 74,52±8,39 | + |
| 15.08.2014 | 28,50±0,00 | 33,00±0,00 | + | 37,88±4,14 | 158,38±26,09 | + |
| 19.08.2014 | - | - | - | 12,02±1,32 | 58,70±11,91 | + |
| 23.08.2014 | - | - | - | 26,60±3,20 | 55,50±14,25 | + |
| 27.08.2014 | - | - | - | 10,91±1,19 | 34,05±9,69 | + |
| 07.09.2014 | 53,83±18,31 | 310,00±138,60 |  | 26,51±4,70 | 82,06±27,02 | + |
| 13.09.2014 | - | - | - | 35,15±4,05 | 150,00±30,00 | + |
| 17.09.2014 | 10,05±0,00 | 8,00±0,00 |  | 22,97±6,90 | ±61,50116,93 | + |
| 21.09.2014 | - | - | - | 21,64±3,05 | 84,68±26,47 | + |
| 25.09.2014 | 38,70±0,00 | 273,00±0,00 |  | 65,55±5,41 | 366,84±50,51 | + |
| 29.09.2014 | - | - | - | 17,41±4,05 | 25,50±14,21 | + |

Number of embryos in the studied colonies was surprisingly low (probably due to exposition of samples to light before fixation) so, we decided not to consider it as reflection of reproductive input marking just their presence/absence.

**Table 3.** Number of studied ovaries of *Celleporella hyalina* (collected in 2013 and 2015).

| **Sampling date** | **Number of colonies** | **Number of ovaries** |
| --- | --- | --- |
| 01.07.13 | 1 | 25 |
| 11.07.13 | 1 | 16 |
| 23.07.13 | 1 | 5 |
| 31.07.13 | 1 | 5 |
| 19.08.15 | 1 | 27 |
